# RTEL1 Regulates G4/R-Loops to Avert Replication-Transcription Collisions

**DOI:** 10.1016/j.celrep.2020.108546

**Published:** 2020-12-22

**Authors:** Panagiotis Kotsantis, Sandra Segura-Bayona, Pol Margalef, Paulina Marzec, Phil Ruis, Graeme Hewitt, Roberto Bellelli, Harshil Patel, Robert Goldstone, Anna R. Poetsch, Simon J. Boulton

**Affiliations:** 1 The Francis Crick Institute, 1 Midland Road, London NW1 1AT, UK; 2 UCL Genetics Institute, University College London, Gower Street, London WC1E 6BT, UK

**Keywords:** RTEL1, R-loops, G-quadruplexes, G4-DNA structures, replication stress, transcription, genome instability

## Abstract

Regulator of telomere length 1 (RTEL1) is an essential helicase that maintains telomere integrity and facilitates DNA replication. The source of replication stress in *Rtel1*-deficient cells remains unclear. Here, we report that loss of RTEL1 confers extensive transcriptional changes independent of its roles at telomeres. The majority of affected genes in *Rtel1*^*−/−*^ cells possess G-quadruplex (G4)-DNA-forming sequences in their promoters and are similarly altered at a transcriptional level in wild-type cells treated with the G4-DNA stabilizer TMPyP4 (5,10,15,20-Tetrakis-(N-methyl-4-pyridyl)porphine). Failure to resolve G4-DNAs formed in the displaced strand of RNA-DNA hybrids in *Rtel1*^*−/−*^ cells is suggested by increased R-loops and elevated transcription-replication collisions (TRCs). Moreover, removal of R-loops by RNaseH1 overexpression suppresses TRCs and alleviates the global replication defects observed in *Rtel1*^*−/−*^ and *Rtel1*^*PIP_box*^ knockin cells and in wild-type cells treated with TMPyP4. We propose that RTEL1 unwinds G4-DNA/R-loops to avert TRCs, which is important to prevent global deregulation in both transcription and DNA replication.

## Highlights

• Rtel1 loss and G4 stabilization cause overlapping transcriptional changes
• Rtel1 loss and G4 stabilization cause transcription-replication conflicts
• Replication stress caused by Rtel1 loss or RTEL1/PCNA mutation is R-loop dependent
• G4 stabilization causes replication stress that is R-loop dependent

## Graphical Abstract

**Figure:**
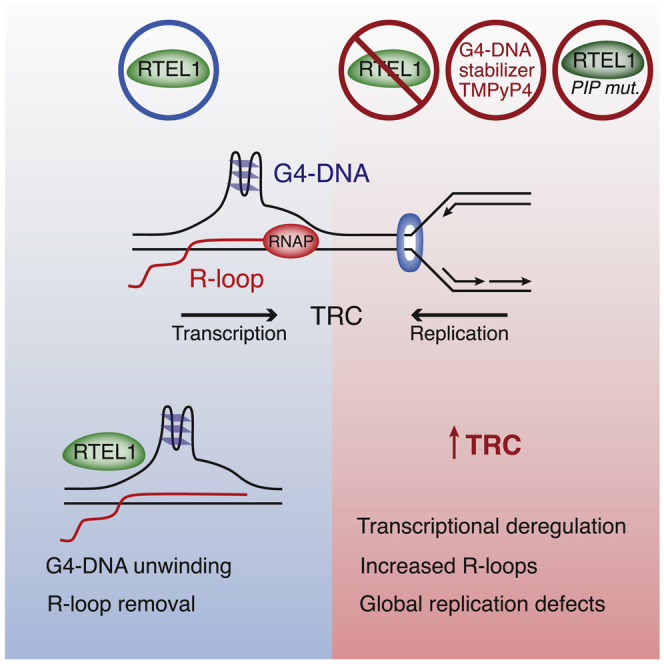

## Teaser

Kotsantis et al. report that loss of the helicase RTEL1 leads to extensive transcriptional changes that overlap with those caused by G4 stabilization. Genome-wide replication stress caused by RTEL1 loss, RTEL1/PCNA mutation, and G4 stabilization is associated with R-loop-dependent transcription-replication conflicts.

## Introduction

Maintenance of genome stability is essential for organismal development and tumor avoidance. A major source of toxic DNA lesions in cells arise from obstacles that interfere with DNA replication and transcription. One such obstacle is the G-quadruplex (G4)-DNA secondary structure, which can form in G-rich repetitive DNA sequences. Mechanisms that resolve G4-DNA structures have been shown to be essential for maintenance of both genome and epigenetic stability. When formed on the leading strand, G4-DNA impedes replication fork progression and hinders DNA replication through repetitive telomeric sequences, leading to telomere fragility (Schiavone et al., 2014, 2016; Vannier et al., 2012).

Transcribed G4-DNA loci often co-exist with stable RNA-DNA hybrids (R-loops) that occur when the nascent RNA molecule hybridizes with the template DNA strand, resulting in G4-DNAs in the displaced single-stranded DNA (ssDNA) (Duquette et al., 2004; Yadav et al., 2016). Evidence for the interdependence between G4-DNA and R-loops comes from observations that R-loops are enriched in sequences harboring G4-DNA motifs in the non-template DNA strand and that R-loop-specific DNA damage is induced by long tandem G-rich repeats and G4-stabilizing ligands (Chen et al., 2019; De Magis et al., 2019; Ginno et al., 2012; Nguyen et al., 2017a). These G-rich promoter sequences can also harbor G4-DNAs that can affect gene regulation and mRNA translation (Varshney et al., 2020). The presence of persistent G4/R-loops may also increase collisions between replication and transcriptional machineries, leading to deleterious transcription-replication conflicts (TRCs). Hence, cells require mechanisms to tolerate, prevent, and resolve TRCs caused by persistent G4/R-loops, most of which remain poorly understood.

Regulator of telomere length 1 (RTEL1) was first identified as impacting telomere length in mice (Ding et al., 2004). *Rtel1* knockout cells exhibit chromosomal aberrations and telomere dysfunction. RTEL1 was also independently identified as anti-recombinase, which disassembles D-loops to counteract non-productive recombination events or reverses homologous recombination (HR) to alter the outcome of the repair reaction (Barber et al., 2008). Based on its D-loop-disrupting activity, it was postulated that telomere dysfunction in *Rtel1*-deficient mouse cells might reflect a failure to dismantle t-loops, which form when the 3′ single-stranded telomeric overhang invades into upstream telomere repeats forming a D-loop intermediate at the point of strand invasion. Indeed, cells lacking RTEL1 fail to efficiently unwind t-loops, which triggers catastrophic processing of persistent t-loops by the SLX1/4 nuclease complex, leading to critically short telomeres (Vannier et al., 2012).

In addition to its roles at vertebrate telomeres, RTEL1 also associates with proliferating cell nuclear antigen (PCNA) via a PIP box domain in its C terminus (Vannier et al., 2013). Although RTEL1 is not constitutively associated with the replisome, it does accumulate at sites of replication stress in a PIP-box-dependent manner, and cells lacking RTEL1 are hyper-sensitive to lesions that stall the replisome. At an organism level, RTEL1-PIP box knockin mice are viable, but aging studies of these mice revealed that RTEL1 acts as a tumor suppressor and is associated with heighted predisposition to lymphoma and medulloblastoma (Vannier et al., 2013). Subsequent genome-wide association studies identified RTEL1 as a susceptibility locus for astrocytomas, high-grade gliomas, and many other cancers. Hypomorphic mutations in human *RTEL1* are also causal for Hoyeraal-Hreidarsson syndrome (HHS), a severe disorder associated with inter-uterine growth retardation, microcephaly, bone marrow failure, immunodeficiency, and many other complications (for review, see Vannier et al., 2014).

While the etiology of HHS remains to be fully elucidated, patient-derived cells and PIP box knockin mouse cells present with both telomeric attrition, increased replication stress, and reduced proliferative capacity in culture. Consistent with a role in facilitating DNA replication, *Rtel1*-deficient and PIP box knockin cells exhibit reduced bromodeoxyuridine (BrdU) incorporation, replication fork asymmetry, reduced replication fork extension rates, and increased origin usage (Vannier et al., 2013). Blocking new origin activation in these cells restored inter-origin distances and fork speeds to wild-type (WT) levels but failed to rescue replication fork asymmetry. Hence, it was proposed that the primary replication defect in *Rtel1*-deficient cells occurs at the level of replication fork stalling and/or collapse (Vannier et al., 2013). More recently, it was reported that loss of *Rtel1* is synthetic lethal with depletion of replication initiation factors, including DNA polymerase epsilon (Bellelli et al., 2020). Collectively, these studies implicate RTEL1 in maintaining telomere homeostasis and facilitating genome-wide DNA replication. However, little is currently known about the source of replication fork stalling and/or collapse in *Rtel1*-deficient cells.

In this study, we show that *Rtel1*-deficient cells exhibit profound changes in transcription with the majority of affected genes possessing G4-DNA-forming sequences in their promoters and many corresponding to chromosomal fragile sites. Moreover, very similar transcriptional changes are observed in WT cells treated with the G4-stabilizing drug TMPyP4. Consistent with studies showing that G4-DNA structures can assemble in the displaced strand formed by R-loops (Duquette et al., 2004; Yadav et al., 2016), we found that loss of RTEL1 results in increased R-loop levels and elevated TRCs. Strikingly, removal of R-loops by RNaseH1 (RNH1) overexpression suppressed the TRCs, global replication defects, and associated genome instability in both *Rtel1*-deficient cells and in WT cells treated with TMPyP4. Based on these data, we propose that RTEL1 unwinds G4-DNA/R-loops to avert TRCs, which we propose are the source of altered transcription and genome-wide replication defects in *Rtel1*-deficient cells.

## Results

### *Rtel1* Deletion and G4 Stabilization Change Transcriptional Landscape in a Similar Pattern

**Figure 1:**
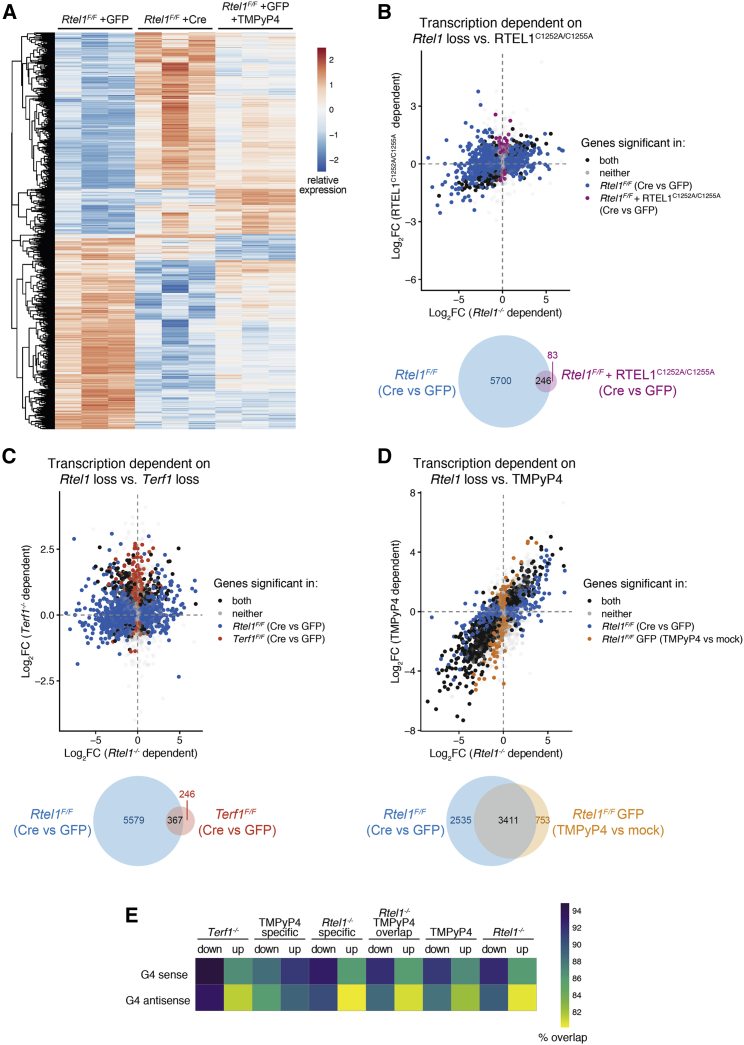
*Rtel1* Deletion Induces Transcriptional Changes That Are Independent of Its Role in Telomere Maintenance and That Overlap with Those Caused by G4 Stabilization (A) RTEL1^F/F^ MEFs were infected with GFP or Cre-GFP adenovirus and collected after 96 h or infected with GFP adenovirus for 48 h, treated with TMPyP4, and collected after 48 h. RNA was isolated, and gene expression levels were analyzed. Heatmap of norm transformed counts per significantly deregulated gene (p < 0.01 in any comparison between groups). Data are scaled by row and clustered with hierarchical clustering. (B) Top: comparative differential gene expression between samples when *Rtel1* was deleted (*Rtel1*^*F/F*^, Cre versus GFP) or contained a t-loop unwinding defect (*Rtel1*^*F/F*^ + RTEL1^C1252A/C1255A^, Cre versus GFP). Differentially expressed genes are differentiated by their significance (p < 0.01) in the respective comparisons. Bottom: Venn diagram of differentially expressed genes. (C) Top: comparative differential gene expression between samples when *Rtel1* was deleted (*Rtel1*^*F/F*^, Cre versus GFP) or are shelterin defective (*Terf1*^*F/F*^, Cre versus GFP). Bottom: Venn diagram of differentially expressed genes. (D) Top: comparative differential gene expression between samples when *Rtel1* was deleted (*Rtel1*^*F/F*^, Cre versus GFP) or treated with TMPyP4 (*Rtel1*^*F/F*^ GFP, TMPyP4 versus mock). Bottom: Venn diagram of differentially expressed genes. (E) Heatmap of comparative proportions of G4-containing promoters in sense and antisense of differentially regulated genes following various treatments as indicated.

To investigate the source of replication aberrations in *Rtel1*-deficient cells, we used an established *Rtel1*^*F/F*^ Cre-lox system to conditionally inactivate *Rtel1* in mouse embryonic fibroblasts (MEFs). Infection of cells with a Cre-GFP-expressing adenovirus, but not a GFP-expressing control, results in loss of the floxed *Rtel1* allele and the subsequent elimination of RTEL1 protein (Vannier et al., 2012). Defects in DNA replication past G4-DNA structures have been linked to epigenetic changes that affect transcription (Sarkies et al., 2010, 2012). Although there has been no prior suggestion of a role for RTEL1 in transcription, we reasoned that failure to unwind G4-DNA structures in the absence of RTEL1 may result in increased TRCs and perturbations in transcription. To test this possibility, we conducted RNA sequencing (RNA-seq) analysis of *Rtel1*-proficient (+GFP) and -deficient (+Cre) cells and examined potential changes in the transcriptional landscape. This analysis revealed that loss of RTEL1 (*Rtel1*^*F/F*^ +Cre) results in substantial changes in the expression levels of 5,698 transcripts with an adjusted p (adj-p) value cutoff of 0.01, with 2,994 transcripts showing increased levels and 2,704 showing reduced levels, relative to control cells (*Rtel1*^*F/F*^ +GFP) (Figures 1A and S1A). While a proportion of these changes could reflect an indirect consequence of *Rtel1*-dependent telomere phenotypes, analysis of telomere dysfunction using an RTEL1^C1252A/C1255A^ mutant, which is defective for t-loop unwinding, but does not result in global/telomere replication defects, or telomere fragility caused by shelterin gene *Terf1* knockout, did not cause such severe transcriptional changes (Figures 1B and 1C) (Sarek et al., 2015; Sfeir et al., 2009). Importantly, we failed to detect significant overlap in genes deregulated between RTEL1^C1252A/C1255A^ or *Terf1*^*−/−*^ and *Rtel1*^*−/−*^_._

Gene Ontology (GO) analysis of affected transcripts following *Rtel1* deletion corroborated that with the exception of the enrichment of checkpoint signaling signatures, which could result indirectly from *Rtel1* loss, the remaining enriched GO categories did not belong to specific biological processes predicted to be affected by attenuated DNA replication, telomere dysfunction, or induction of the DNA damage response (Figures S1B and S1C). GO analysis of transcriptional changes revealed similar results between *Rtel1* deletion and TMPyP4 treatment (Figures S1D and S1E). Further investigation of the distinct groups of GO analysis of *Rtel1* deletion and TMPyP4 treatment did not detect any meaningful enrichment of GOs. We thus considered that RTEL1, by virtue of its ability to unwind G4-DNA structures (Vannier et al., 2013), could directly affect the transcriptional landscape.

We next evaluated whether the effect of a G4-DNA-stabilizing ligand, TMPyP4, could phenocopy the transcriptional changes associated with *Rtel1* deficiency. Indeed, strikingly similar transcriptional changes to those seen in *Rtel1*-deficient cells were observed in WT cells treated with TMPyP4 (10 μM) (*Rtel1*^*F/F*^ +GFP +TMPyP4) (Figures 1A and 1D). Despite the majority of promoters possessing potential G4-DNA-forming sequences both in sense and antisense strands, we observed a higher overlap between the RTEL1/TMPyP4 differentially expressed genes and those harboring a G4-DNA-forming sequence in the sense strand, raising the possibility that G4-DNA structures may form in the displaced ssDNA of R-loops that form co-transcriptionally, in *cis* (Figures 1E and S1G). Together, these results suggest that while the transcriptional changes seen in *Rtel1*-deficient cells are largely independent of RTEL1’s role at telomeres and are not caused by telomere fragility or loss, *Rtel1* deletion or G4 stabilization by TMPyP4 alters the transcriptional landscape in a very similar manner, with the majority of affected genes containing G4-DNA-forming sequences in their promoter.

### *Rtel1* Deletion Affects Fragile Sites and Increases R-Loop Levels

**Figure 2:**
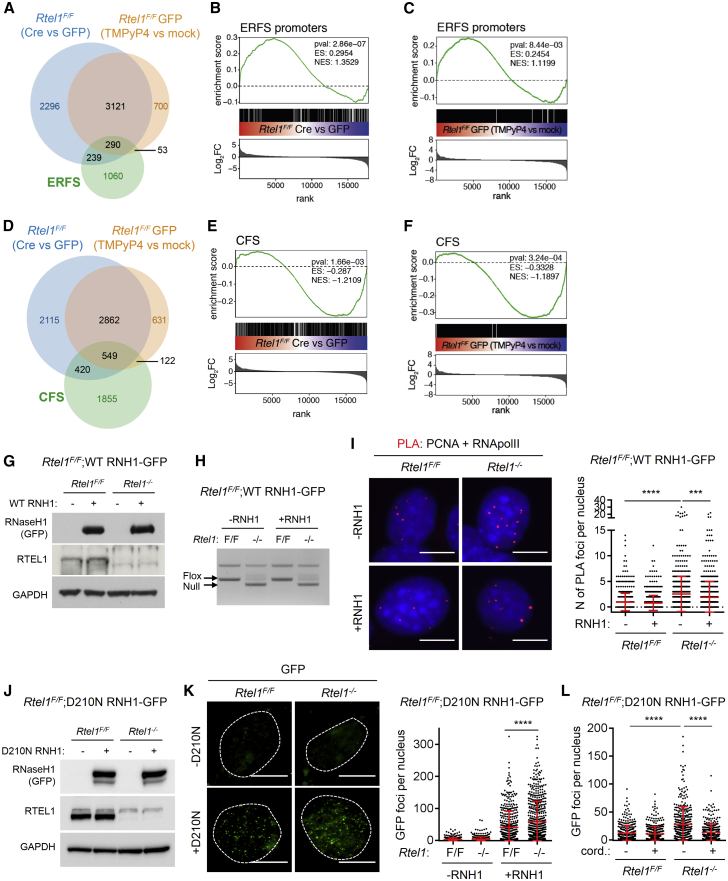
RTEL1 Counteracts Transcription-Replication Conflicts by Regulating R-Loops (A) Venn diagram of differentially regulated genes of samples with deleted *Rtel1* (*Rtel1*^*F/F*^, Cre versus GFP) or samples treated with TMPyP4 (*Rtel1*^*F/F*^ GFP, TMPyP4 versus mock), and their overlap with ERFSs. (B) GSEA that shows enrichment of genes with ERFSs in their promoters. Genes are ranked dependent of logarithm of fold change (Log_2_FC) of differential expression with deleted *Rtel1* (*Rtel1*^*F/F*^, Cre versus GFP), and the overall enrichment score (ES) and normalized enrichment score (NES) with the respective p value (pval) of the enrichment were determined. (C) GSEA that shows enrichment of genes with ERFSs in their promoters. Genes are ranked dependent of Log_2_FC of differential expression upon TMPyP4 treatment, and the overall ES and NES with the respective pval of the enrichment were determined. (D) Venn diagram of differentially regulated genes of samples with deleted *Rtel1* (*Rtel1*^*F/F*^, Cre versus GFP) or treated with TMPyP4 (*Rtel1*^*F/F*^ GFP, TMPyP4 versus mock), and their overlap with CFSs. (E) GSEA that shows enrichment of genes overlapping with CFSs. Genes are ranked dependent of Log_2_FC of differential expression with deleted *Rtel1* (*Rtel1*^*F/F*^, Cre versus GFP), and the overall ES and NES with the respective pval of the enrichment were determined. (F) GSEA that shows enrichment of genes with CFSs in their promoters. Genes are ranked dependent of Log_2_FC of differential expression upon TMPyP4 treatment, and the overall ES and NES with the respective pval of the enrichment were determined. (G) *Rtel1*^*F/F*^;WT RNH1-GFP MEFs were infected with GFP or Cre-GFP adenovirus. After 48 h, doxycycline was added, and cells were collected after 48 h. The cells were then lysed, and whole-cell extracts were analyzed by SDS-PAGE and immunoblotted for GFP, RTEL1, and glyceraldehyde 3-phosphate dehydrogenase (GAPDH). (H) Cells were treated as in (G), and genomic DNA was isolated and loss of *Rtel1* was verified by PCR. (I) Cells were treated as in (G), and the interaction between PCNA and RNA polymerase II (RNApolII) was assessed by PLA. Left: representative images of PLA. Right: quantification of PLA. Data are represented as mean ± SD (n = 4). (J) *Rtel1*^*F/F*^;D210N RNH1-GFP MEFs were infected with red fluorescent protein (RFP) or improved Cre-RFP (iCre-RFP) adenovirus. After 48 h, doxycycline was added, and cells were collected after 48 h. The cells were then lysed, and whole-cell extracts were analyzed by SDS-PAGE and immunoblotted for GFP, RTEL1, and GAPDH. (K) Cells were treated as in (J) and immunostained for GFP. Left: representative images of GFP immunofluorescence. Right: quantification of RNaseH1^D210N^-GFP foci per nucleus of cells. Data are represented as mean ± SD (n = 3). (L) *Rtel1*^*F/F*^;D210N RNH1-GFP MEFs were infected with RFP or iCre-RFP adenovirus. After 96 h, cells were treated with cordycepin for 3.5 h and immunostained for GFP. Quantification of RNaseH1^D210N^-GFP foci per nucleus of cells. Data are represented as mean ± SD (n = 3). The pvals were determined by unpaired t test, with ^∗∗∗^p < 0.001 and ^∗∗∗∗^p < 0.0001. Scale bars, 10 μm.

Inactivation of RTEL1 is known to lead to increased chromatid breaks on metaphase chromosomes, but the source of these breaks is unclear. Prompted by the severe effects of *Rtel1* knockout on DNA replication efficiency (Vannier et al., 2013) and transcription, we considered whether the absence of RTEL1 would lead to TRCs at chromosomal fragile sites, which are susceptible to breakage upon replication stress, thus affecting transcription. We first analyzed the effect of *Rtel1* loss or TMPyP4 treatment on early replicating fragile sites (ERFSs), which occur in gene-rich regions, are transcriptionally active, and coincide with an increased density of replication origins (Barlow et al., 2013). Of the total 12,118 defined ERFSs, 1,642 overlap with promoters, and 529 (32.2%) of those 1,642 were significantly affected by *Rtel1* deletion, while 343 (20.9%) were affected upon G4 stabilization (Figure 2A). Gene set enrichment analysis (GSEA) revealed that upregulation of these genes upon *Rtel1* loss or TMPyP4 treatment is highly significant (Figures 2B and 2C).

Next, we focused on common fragile sites (CFSs), sites prone to breakage upon replication obstruction that have been mapped cytologically on mitotic chromosomes and are associated with large genes, long A-T repeat regions, and incomplete DNA replication (for review, see Glover et al., 2017). Similar to ERFSs, 32.9% (969/2,946) of the defined CFSs were differentially transcribed in the absence of *Rtel1* and 22.8% (671/2,946) of CFSs were differentially expressed due to G4 stabilization (Figure 2D). Strikingly, the CFS gene signature was significantly downregulated in TMPyP4-treated and *Rtel1*-deficient cells (Figures 2E and 2F). Thus, despite transcriptional changes present upon *Rtel1* loss overlapping with both ERFSs and CFSs, genes located at such fragile sites exhibit non-random effects on transcription, with upregulation observed at ERFSs and downregulation at CFSs.

Chromosomal fragile sites are well-established hotspots for TRCs and R-loops formation (Helmrich et al., 2011). We therefore considered the possibility that the replication and transcription defects observed in *Rtel1*-deficient cells may result from a failure to unwind G4-DNA residing within R-loops, which may lead to TRCs. To test this hypothesis, we first generated *Rtel1*^*F/F*^ MEFs that upon addition of doxycycline (2 μg/mL) overexpress WT RNaseH1-GFP, which degrades RNA-DNA hybrids and leads to the removal of R-loops. As previously described, infecting cells with Cre-GFP adenovirus, but not with the GFP control, removes the floxed *Rtel1* allele and eliminates RTEL1 protein (Figures 2G and 2H). Overexpression of WT RNaseH1-GFP reduced R-loop levels in selected genomic regions as assessed by DNA-RNA immunoprecipitation (DRIP)-qPCR (Figures S2A and S2B), had no effect on the cell-cycle profile 96 h after induction, and all cells were GFP positive (Figures S2C–S2E). Using an established proximity ligation assay (PLA) to detect TRCs (Hamperl et al., 2017), we observed a significant increase in the frequency of PCNA-RNA polymerase II interactions in *Rtel1*-deficient cells, but not in controls (Figures 2I, S2F, and S2G). Since TRCs may arise as a result of a failure to resolve R-loops, we tested whether TRCs in RTEL1-deleted cells are R-loop dependent. Indeed, overexpression of WT RNaseH1-GFP reduced the number of TRCs in *Rtel1*-deficient cells (Figure 2I), indicating that persistent R-loops are responsible for the elevated TRCs in this context.

To further examine an involvement of RTEL1 in R-loop metabolism, we created *Rtel1*^*F/F*^ MEFs that upon addition of doxycycline overexpress the catalytically dead RNaseH1^D210N^-GFP mutant, which binds to R-loops without degrading them and can be used to directly visualize R-loops in cells (Chen et al., 2019) (Figure 2J). Previous reports have shown that RNaseH1^D210N^-GFP accumulates in distinct foci in response to replication stress (Chappidi et al., 2020). Treatment with aphidicolin, which leads to R-loop accumulation (Hamperl et al., 2017), induced RNaseH1^D210N^-GFP foci formation (Figures S2H and S2I). Deletion of *Rtel1* alone also resulted in a significant increase in the number of RNaseH1^D210N^-GFP foci per nucleus (Figures 2K). Treating cells with cordycepin, which blocks transcription elongation, abolished induction of RNaseH1^D210N^-GFP foci in cells lacking RTEL1 (Figures 2L and S2J). Taken together, these data suggest that RTEL1 suppresses R-loop-dependent TRCs, which could reflect a role in unwinding G4-DNA structures that reside within R-loops.

### *Rtel1* Deletion Causes R-Loop-Dependent Genome-wide Replication Stress

**Figure 3:**
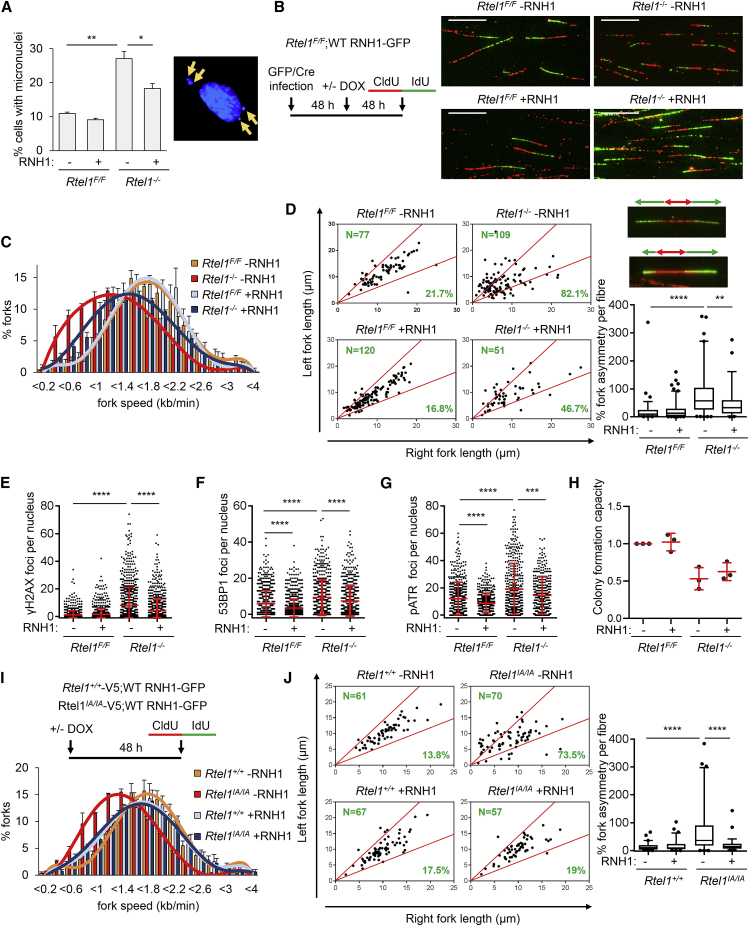
*Rtel1* Deletion Causes Genome-wide R-Loop-Dependent Replication Stress (A) *Rtel1*^*F/F*^;WT RNH1-GFP MEFs were infected with GFP or Cre-GFP adenovirus. After 48 h, doxycycline was added, and cells were collected after 48 h. The cells were then fixed, and the percentage of cells with >1 micronucleus was quantified. Left: quantification. Right: representative images of micronuclei. Data are represented as mean ± SE (n = 3). (B) *Rtel1*^*F/F*^;WT RNH1-GFP MEFs were treated as in (A), and a DNA fiber assay was performed. Left: experimental setup. Right: representative images of DNA fibers. (C) Distribution of replication fork speeds of DNA fibers as prepared in (B). Data are represented as mean ± SE (n = 3). (D) Left: scatterplot of fork asymmetry of DNA fibers as prepared in (B). Right, top: representative images of symmetric and asymmetric DNA fibers. Right, bottom: quantification of fork asymmetry of DNA fibers as prepared in (B) (n = 4). (E) *Rtel1*^*F/F*^;WT RNH1-GFP MEFs were treated as in (A) and immunostained for γH2AX. Quantification of γH2AX foci per nucleus. Data are represented as mean ± SD (n = 3). (F) *Rtel1*^*F/F*^;WT RNH1-GFP MEFs were treated as in (A) and immunostained for 53BP1. Quantification of 53BP1 foci per nucleus. Data are represented as mean ± SD (n = 3). (G) *Rtel1*^*F/F*^;WT RNH1-GFP MEFs were treated as in (A) and immunostained for pATR-S428. Quantification of pATR-S428 foci per nucleus. Data are represented as mean ± SD (n = 3). (H) Colony formation capacity in *Rtel1*^*F/F*^;WT RNH1-GFP MEFs infected with GFP or Cre-GFP adenovirus with or without doxycycline. Data are mean ± SD normalized to GFP and 0 μg/mL doxycycline condition (n = 3). (I) *Rtel1*^*+/+*^-V5;WT RNH1-GFP and *Rtel1*^*IA/IA*^-V5;WT RNH1-GFP were treated with doxycycline for 48 h, and a DNA fiber assay was performed. Top: experimental setup. Bottom: distribution of replication fork speeds of DNA fibers. Data are represented as mean ± SE (n = 3). (J) Left: scatterplot of fork asymmetry of DNA fibers prepared as in (I). Right, bottom: quantification of fork asymmetry of DNA fibers prepared as in (I) (n = 3). In boxplots, horizontal line denotes the mean; whiskers denote the 5th and 95th percentiles. The pvals were determined by unpaired t test, with ^∗^p < 0.05, ^∗∗^p < 0.01, ^∗∗∗^p < 0.001, and ^∗∗∗∗^p < 0.0001. Scale bars, 10 μm.

Prompted by these findings, we hypothesized that the increased replication fork stalling and/or collapse observed in the absence of RTEL1 may reflect TRCs arising at R-loops. If this hypothesis is correct, we reasoned that removing R-loops by overexpressing WT RNaseH1-GFP should suppress the replication defects in *Rtel1*-deficient cells. Consistent with this possibility, the increased incidence of micronuclei that form in the absence of RTEL1 was reduced upon WT RNaseH1-GFP overexpression (Figure 3A), which also restored the rate of replication fork progression in *Rtel1*-deficient cells to levels approaching WT controls (Figures 3B and 3C). Similarly, replication fork asymmetry, reflective of replication fork stalling and/or collapse in *Rtel1*-deficient cells, was also suppressed by WT RNaseH1-GFP overexpression (Figure 3D). The suppression of replication stress in *Rtel1*-deficient cells by WT RNaseH1-GFP is independent of changes in the cell cycle, which remained unaffected throughout the experiments (Figure S3A). Importantly, overexpression of the catalytically dead RNaseH1^D210N^-GFP mutant did not rescue fork progression, fork asymmetry, or micronuclei in *Rtel1*-deleted cells (Figures S3B–S3D).

Heightened replication stress in the absence of RTEL1 also manifests as increased phosphorylation of histone H2AX (γH2AX) and induction of p53 binding protein 1 (53BP1) foci, a double-strand break marker. Overexpression of WT RNaseH1-GFP reduced the levels of γH2AX and 53BP1 foci (Figures 3E, 3F, S3E, and S3F). Replication protein A (RPA) is a ssDNA-binding protein that was recently shown to colocalize with R-loops (Nguyen et al., 2017b). *Rtel1*-deficient cells exhibited increased levels of RPA foci, and this was alleviated upon overexpression of WT RNaseH1-GFP (Figures S3H and S3I). Ataxia telangiectasia and Rad3 related (ATR) kinase is a master regulator of the replication stress response and has been shown to be activated by head-on TRCs (Hamperl et al., 2017). Loss of *Rtel1* activated the ATR pathway as shown by increased nuclear phosphorylated at Serine 428 ATR (pATR S428) foci, and this was suppressed by overexpression of WT RNaseH1-GFP (Figures 3G and S3G).

**Figure 4:**
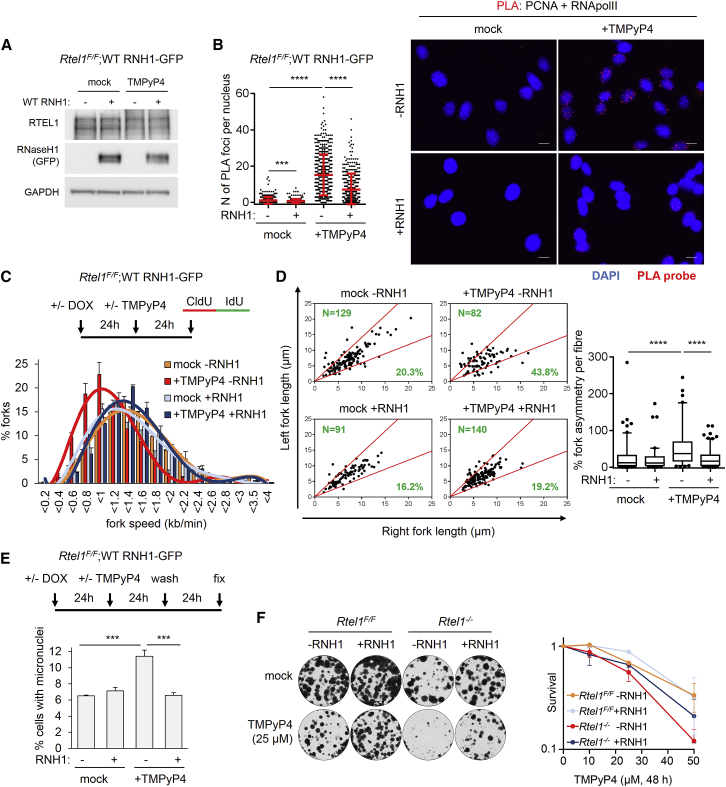
G4-DNA Stabilization Causes Genome-wide R-Loop-Dependent Replication Stress (A) *Rtel1*^*F/F*^;WT RNH1-GFP MEFs were treated with doxycycline. After 24 h, TMPyP4 was added for 24 h. Cells were then collected and lysed, and whole-cell extracts were analyzed by SDS-PAGE and immunoblotted for GFP, RTEL1, and GAPDH. (B) *Rtel1*^*F/F*^;WT RNH1-GFP MEFs were treated as in (A), and the interaction between PCNA and RNApolII was assessed by PLA. Right: representative images of PLA. Left: quantification of PLA (n = 3). (C) *Rtel1*^*F/F*^;WT RNH1-GFP MEFs were treated as in (A), and DNA fiber assay was performed. Top: experimental setup. Bottom: distribution of replication fork speeds of DNA fibers. Data are represented as mean ± SE (n = 3). (D) Left: scatterplot of fork asymmetry of DNA fibers as prepared in (C). Right, bottom: quantification of fork asymmetry of DNA fibers as prepared in (C). In boxplots, horizontal line denotes the mean; whiskers denote the 5th and 95th percentiles (n = 3). (E) *Rtel1*^*F/F*^;WT RNH1-GFP MEFs were treated with doxycycline. After 24 h, TMPyP4 was added for 24 h and then removed. Cells were incubated for another 24 h and then fixed. The percentage of cells with micronuclei was quantified. (F) Right: TMPyP4 colony survival assay in *Rtel1*^*F/F*^;WT RNH1-GFP MEFs. Data are mean ± SEM normalized to untreated cells (n = 3). Left: representative images of colonies. The pvals were determined by unpaired t test, with ^∗∗∗^p < 0.001 and ^∗∗∗∗^p < 0.0001. Scale bars, 10 μm.

Having established that the induction of R-loops in the absence of *Rtel1* is transcription dependent (Figures 2L), we next tested whether active transcription drives replication stress in *Rtel1*-deficient cells. Transient transcription inhibition fully rescued *Rtel1* loss-induced γH2AX foci (Figures S3J), which further suggests that *Rtel1* loss-induced replication stress is caused by R-loops formed during transcription. Furthermore, overexpression of WT RNaseH1-GFP led to a small but significant increase in colony formation capacity of *Rtel1*-depleted MEFs, which is not as pronounced in colony number but is highly significant for colony size, which is dependent on cellular proliferation once colonies are formed (Figures 3H and 4F).

Since RTEL1’s ability to regulate DNA replication is dependent on its interaction with PCNA, we next investigated the role of this interaction in counteracting R-loops. By overexpressing WT RNaseH1-GFP in RTEL1^+/+^-V5 as well as in RTEL1^IA/IA^-V5 MEFs, we were able to rescue both the fork slowing and fork asymmetry defects in RTEL1^IA/IA^-V5 MEFs (Figures 3I, 3J, and S4A). Preventing the interaction between RTEL1 and telomere repeat binding factor 2 (TRF2) via a mutation in the RTEL1 C4C4 motif hinders telomere recruitment and t-loop unwinding, but it does not confer replication stress (Sarek et al., 2015). Expression of the RTEL1^C1252A/C1255A^ mutant alone did not induce RNaseH1^D210N^-GFP foci formation, which suggests that RTEL1 recruitment to telomeres is not involved in R-loop regulation (Figures S4B). Overall, our findings suggest that replication stress caused by *Rtel1* deletion and more specifically, loss of its interaction with PCNA, arise due to persistent G4/R-loops.

### Telomeric Stress Caused by *Rtel1* Deletion Is R-Loop Independent

Apart from its effect on genome-wide replication, RTEL1 is critical for maintaining telomere integrity (Sarek et al., 2015, 2019; Vannier et al., 2012). In particular, *Rtel1* loss causes telomeric fragility, which is indicative of telomere replication defects, and telomeric shortening and loss, which is caused by inefficient t-loop unwinding and aberrant processing. We next examined the link between R-loops and RTEL1 in telomere maintenance. Notably, overexpression of WT RNaseH1-GFP on its own caused telomeric fragility (Figures S4C and S4D). This may be due to telomeric repeat-containing RNA (TERRA) hybrids residing at telomeres, which have been shown to be regulated by RNaseH1 (Arora et al., 2014). To test this hypothesis, we performed immunofluorescence (IF)/TERRA fluorescence *in situ* hybridization (FISH) and found that deletion of *Rtel1* increases TERRA foci at telomeres (Figures S4F–S4H). Since these foci are resistant to RNaseA treatment, they may be related to RNA-DNA hybrids at telomeres. In combination with *Rtel1* loss, overexpression of WT RNaseH1-GFP failed to rescue telomeric fragility (Figures S4C and S4D). Furthermore, WT RNaseH1-GFP overexpression increased the frequency of telomere loss events observed upon *Rtel1* deletion (Figures S4C and S4E). Taken together, these findings show that telomeric dysfunction caused by loss of RTEL1 is not caused by persistent R-loops; rather, removing R-loops that potentially involve TERRA exacerbates this phenotype.

### G4-DNA Stabilization Causes R-Loop-Induced Genome-wide Replication Stress

Since G4-DNA stabilization increases R-loops at regions harboring predicted G4 sequences (De Magis et al., 2019), we hypothesized that G4/R-loops might create an obstacle to replication fork progression and lead to genome-wide replication stress. To test this hypothesis, we first assessed the ability of TMPyP4 to induce R-loops. Imaging analysis of RNaseH1^D210N^-GFP revealed that TMPyP4 treatment decreased the number of foci but caused a significant increase in the overall nuclear levels of RNaseH1^D210N^-GFP signal, which was only mildly increased when *Rtel1* was deleted (Figures S5A–S5C). The reduction of TMPyP4-induced RNaseH1^D210N^-GFP foci could be attributed to the fact that RNaseH1^D210N^-GFP may bind less strongly to G4/R-loops when also bound by TMPyP4. Titration experiments at different time points following TMPyP4 treatment revealed that G4-DNA stabilization causes early induction of RNaseH1^D210N^-GFP foci that are fewer and smaller than the foci caused by *Rtel1* deletion (Figure S5D). Moreover, induction of TMPyP4-induced RNaseH1^D210N^-GFP foci precedes DNA damage as assessed by γH2AX IF (Figure S5E). Colocalization with PCNA revealed that 66% of the TMPyP4-induced RNaseH1^D210N^-GFP foci reside within S-phase. Finally, TMPyP4-induced RNaseH1^D210N^-GFP signal is unrelated to RTEL1 levels as RTEL1 is still present upon TMPyP4 treatment (Figure 4A). Nevertheless, chromatin-bound RTEL1-V5 is induced upon TMPyP4 treatment (Figure S5H), which suggests a potential response to increased G4/R-loop levels.

Prompted by our findings that G4 stabilization increases R-loop levels, we tested the possibility that it may cause TRCs. PLA for TRCs revealed that TMPyP4 treatment caused a profound increase in TRCs that was significantly reduced upon overexpression of WT RNaseH1-GFP (Figures 4B), which indicates that TMPyP4-induced TRCs are R-loop dependent. This finding led us to examine the role of R-loops in TMPyP4-induced replication stress in *Rtel1*-proficient cells. TMPyP4 treatment caused fork slowing and asymmetry and increased micronuclei formation that were rescued upon overexpression of WT RNaseH1-GFP (Figures 4C–4E). Finally, *Rtel1* deletion sensitized cells to G4-DNA stabilization by TMPyP4, and this was partially alleviated by overexpression of WT RNaseH1-GFP (Figure 4F).

### *Rtel1*^*−/−*^-Induced R-Loops Are Related to *Rtel1*^*−/−*^-Induced Transcriptional Changes

**Figure 5:**
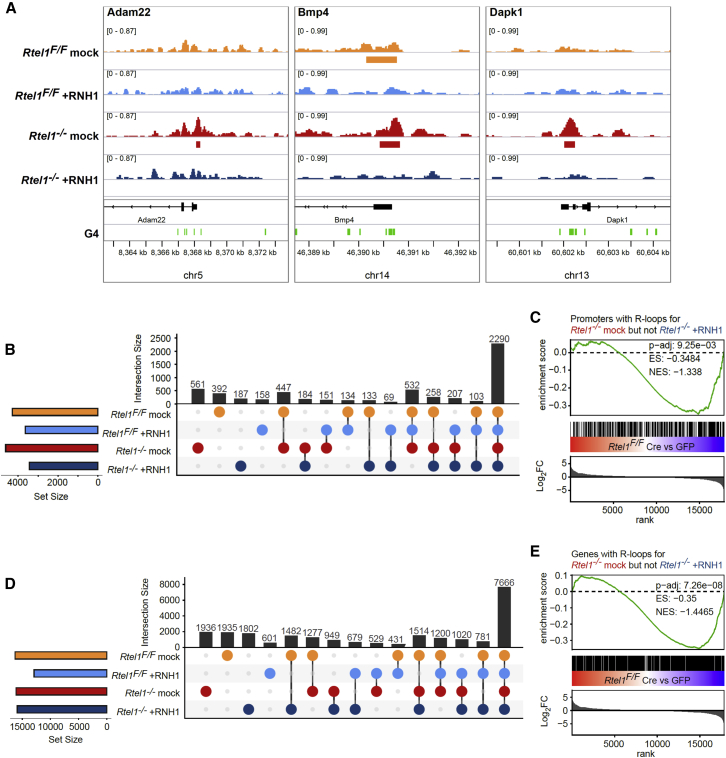
*Rtel1*^*−/−*^-Induced R-Loops Are Related to *Rtel1*^*−/−*^-Induced Transcriptional Changes (A) *Rtel1*^*F/F*^;WT RNH1-GFP MEFs were infected with GFP or Cre-GFP adenovirus. After 48 h, doxycycline was added, and cells were collected after 48 h and used for R-loop detection with DRIP-seq. Genome browser plots of normalized read coverage, called peaks of DRIP-seq, and associated predicted G4 structures in three different genomic locations. (B) Overlap analysis of DRIP-seq peaks in promoters. Upset plot that depicts the numbers of promoters with R-loops shared between *Rtel1*^*F/F*^ and *Rtel1*^*−/−*^, dependent on RNaseH1. (C) GSEA that shows transcriptional enrichment of genes with *Rtel1*^*−/−*^-specific and RNH1-sensitive promoter R-loops. Genes are ranked dependent of Log_2_FC of differential expression with deleted *Rtel1* (*Rtel1*^*F/F*^, Cre versus GFP). The overall ES and NES with the respective pvals were determined of the enrichment with R-loop peaks that are present with *Rtel1* deletion, yet not present with RNaseH1 overexpression. (D) Overlap analysis of DRIP-seq peaks in genes. Upset plot that depicts the numbers of genes with R-loops shared between *Rtel1*^*F/F*^ and *Rtel1*^*−/−*^, dependent on RNaseH1. (E) GSEA that shows transcriptional enrichment of genes with *Rtel1*^*−/−*^-specific and RNaseH1-sensitive R-loops. Genes are ranked dependent of Log_2_FC of differential expression with deleted *Rtel1* (*Rtel1*^*F/F*^, Cre versus GFP). The overall ES and NES with the respective pvals were determined of the enrichment with R-loop peaks that are present with *Rtel1* deletion, yet not present with RNaseH1 overexpression.

To further assess the relationship between the *Rtel1*^*−/−*^-induced R-loops and their effect on transcription, we performed DRIP-seq in *Rtel1*-proficient and -deficient MEFs upon overexpression of WT RNaseH1-GFP and conducted an integrative analysis with our previous RNA-seq-derived data. As shown in genome browser plots, R-loop peaks accumulate at specific promoters due to *Rtel1* loss, which was reduced upon overexpression of WT RNaseH1-GFP (Figures 5A and S5I). Further assessment was focused on the promoters (n = 561) and genes (n = 1,936) that follow this pattern (Figures 5B and 5D). Interestingly, there is an enrichment within genes that are downregulated upon *Rtel1* deletion, both when assessing R-loops containing genes (normalized enrichment score [NES] = −1.4465, p-adj = 7.26e−08) and specifically genes with R-loops at their promoters (NES = −1.338, p-adj = 9.25e−03) (Figures 5C and 5E). These data further support that *Rtel1* deletion causes R-loop accumulation and is associated with transcriptional changes.

## Discussion

In this study, we uncover an unappreciated role for the helicase RTEL1 in counteracting G4/R-loops that impacts on both global transcription and DNA replication. Our data show that deletion of *Rtel1* causes R-loop accumulation and global transcriptional changes that overlap to a significant extent with the changes caused by the G4-DNA stabilizer TMPyP4 in WT cells (Figure 1A). A significant proportion of the affected genes possess predicted G4-DNA-forming sequences in their promoters and are coincident with ERFSs and CFSs (Figures 2A–2F), which are susceptible to breakage caused by R-loop-dependent TRCs. One of the key functions of R-loops is in the positive and negative regulation of transcription. R-loops can protect DNA from methylation, promote recruitment of H3K4me3 (trimethylation at lysine 4 on histone H3) methyltransferases to enable gene activation (Ginno et al., 2012), and may contribute to the formation of H3K9me2 (dimethylation at lysine 9 on histone H3) chromatin marks to facilitate transcriptional termination of a subset of genes (Skourti-Stathaki et al., 2014). Recently, R-loops were reported to form at Polycomb target genes and contribute to gene silencing (Skourti-Stathaki et al., 2019). G4s have been suggested to form in the displaced strand of R-loops, which impacts their stability (Duquette et al., 2004; Yadav et al., 2016) and affects transcriptional regulation. G4s can also regulate transcription by controlling histone mark deposition on DNA (Papadopoulou et al., 2015; Sarkies et al., 2010). Since *Rtel1* deletion leads to R-loop accumulation (Figure 2K) and TRCs (Figure 2I) and is associated with transcriptional downregulation of genes containing R-loops within them and more specifically at their promoters (Figures 5 and S5I), we propose that failure to resolve G4/R-loops is likely responsible for the transcriptional changes observed in this context.

In addition to the impact on transcription, our study reveals that G4/R-loops are the likely source of genome-wide replication defects in *Rtel1*-deficient cells. This is supported by our observation that overexpression of WT RNaseH1-GFP partially rescues the reduced fork extension rates, fork asymmetry, micronuclei, and 53BP1 and γH2AX foci in *Rtel1*^−/−^ (Figures 3A–3F) and *Rtel1*^*PIP_box*^ knockin (Figures 3I, 3J, and S4A) cells. This implies that RTEL1 is important for the removal of R-loops in a PIP-box-dependent manner, which is consistent with a recent paper showing that Poldip3 facilitates RTEL1 recruitment to chromatin to remove R-loops (Björkman et al., 2020). We previous established that increased replication fork stalling and/or collapse is the primary source of replication problems in *Rtel1*^*−/−*^ and *Rtel1*^*PIP_box*^ knockin cells (Vannier et al., 2013). However, the cause of fork stalling and/or collapse was unknown. Our findings and those of others (Björkman et al., 2020; Takedachi et al., 2020; Wu et al., 2020) suggest that the replication defects in *Rtel1*-deficient cells are primarily caused by TRCs resulting from inefficient removal of G4/R-loops. While the precise mechanism by which RTEL1 counteracts G4/R-loops remains to be defined, RTEL1 can unwind telomeric G4s *in vitro* (Vannier et al., 2013), so it could use this activity to remove G4s within a G4/R-loop. RTEL1 is also a potent D-loop unwinding enzyme (Barber et al., 2008), so it could conceivably unwind R-loops, which are structurally similar to D-loops. However, we cannot exclude the possibility that RTEL1 loss causes G4 accumulation randomly throughout the genome that may block the transcription machinery, leading to accumulation of R-loops and subsequent transcriptional changes.

G4 stabilization by TMPyP4 treatment caused an increase in R-loop levels that preceded DNA damage (Figures S5D and S5E), which suggests R-loops are a source of genomic instability in this context. Interestingly, the size and number of R-loops as assessed by RNaseH1^D210N^-GFP foci was much fewer and smaller relative to that observed following *Rtel1* loss (Figures S5A, S5B, and S5D), which may be attributed to TMPyP4 inducing a specific subgroup of G4s in contrast to RTEL1 having a broader effect. Importantly, overexpression of WT RNaseH1GFP rescued replication stress and reduced sensitivity to TMPyP4 (Figure 4). Furthermore, PLA revealed that TMPyP4 treatment causes TRCs that are R-loop dependent (Figures S5B). Therefore, our findings reveal that G4 stabilization causes an accumulation of R-loops that leads to genomic instability through increased TRCs.

Several recent studies have also reported that depletion of RTEL1 leads to the accumulation of G4/R-loops (Björkman et al., 2020; Wu et al., 2020). Notably, Wu et al. (2020) reported that RTEL1 is required for efficient mitotic DNA synthesis (MiDAS) at loci prone to form G4-associated R-loops. In this context, overexpression of WT RNaseH1 was found to reduce MiDAS. This observation, together with our finding that WT RNaseH1-GFP overexpression rescues both TRCs and replication defects in *Rtel1* cells, implies that removing R-loops is a prerequisite for MiDAS activation. Takedachi et al. (2020) reported that RTEL1 interacts with the nuclease scaffold protein SLX4 to prevent TRCs and genome-wide replication stress. Blocking transcription was found to rescue these phenotypes, which we show also abolishes the induction of RNaseH1^D210N^-GFP foci as well as γH2AX in *Rtel1*-deficient cells (Figures 2L and S3J). Taken together, these data establish that removing R-loops by either blocking transcription or by WT RNaseH1 overexpression suppresses TRCs, rescues the deleterious effect of RTEL1 deficiency on replication fork stalling and/or collapse, and suppresses MiDAS. Hence, these observations converge on G4/R-loops as the source of replication stress in *Rtel1*-deficient cells.

In contrast to the suppression of global replication defects in *Rtel1*-deficient cells, WT RNaseH1 overexpression exacerbated telomere dysfunction in the absence of RTEL1 (Figures S4C–S4E). A possible explanation for this may involve the engagement of the non-coding TERRA RNA with telomeric DNA, which is known to form an RNA:DNA hybrid that is recognized and processed by RNaseH1. RNaseH1 has been shown to regulate TERRA levels and impact on the alternative lengthening of telomeres pathway, which maintains telomeres in a subset of cancers cells (Arora et al., 2014). Our data show that *Rtel1* deletion induces TERRA levels (Figures S4F–S4H), so it is possible that the telomeric fragility in *Rtel1*^*+/+*^ cells and the increased telomeric loss in *Rtel1*^*−/−*^ cells upon overexpression of WT RNaseH1-GFP (Figures S4D and S4E) is related to a disturbance in TERRA homeostasis.

In summary, our observations, together with several recent reports from others, suggest that failure to remove G4/R-loops is the primary source of replication stress in *Rtel1*-deficient cells.

## STAR★Methods

### Key Resources Table

**Table:** 

REAGENT or RESOURCE	SOURCE	IDENTIFIER
**Antibodies**		

Goat Anti-Rat IgG (H+L), Alexa Fluor 594 Conjugated	Thermo Fisher	Cat#A-11007; RRID: AB_141374
Rabbit Anti-Mouse IgG (H+L), Alexa Fluor488 Conjugated	Thermo Fisher	Cat#A-11059; RRID: AB_142495
Goat Anti-Rabbit IgG (H+L), Alexa Fluor488 Conjugated	Thermo Fisher	Cat#A-11034; RRID: AB_2576217
Goat Anti-Mouse IgG (H+L), Alexa Fluor594 Conjugated	Thermo Fisher	Cat#A-11005; RRID: AB_2534073
Goat Anti-Rabbit IgG (H+L), Alexa Fluor594 Conjugated	Thermo Fisher	Cat#A-11037; RRID: AB_2534095
Rabbit anti-53BP1	Bethyl Laboratories	Cat#A300-272A; RRID: AB_185520
Mouse monoclonal anti-γH2AX, clone JBW301	Millipore	Cat#05-63; RRID: AB_309864
Mouse monoclonal anti-RPA32/RPA2	Abcam	Cat#ab2175; RRID: AB_302873
Rabbit anti-Phospho-ATR (Ser428)	Cell Signaling Technology	Cat#2853; RRID: AB_2290281
Rabbit anti-RTEL1	Novus Biologicals	Cat#NBP2-22360; RRID: AB_2722642
Rabbit anti-PCNA	Santa Cruz	Cat#SC7907; RRID: AB_2160375
Mouse anti-PCNA	Santa Cruz	Cat#SC56; RRID: AB_628110
Rabbit anti-TRF2	Cell Signaling Technology	Cat#13136; RRID: AB_2722641
Rabbit anti-V5	Sigma-Aldrich	Cat#V8137; RRID: AB_261889
Rabbit anti-GFP	Abcam	Cat#ab290; RRID: AB_303395
Mouse anti-GFP	Roche	Cat#11814460001; RRID: AB_390913
Mouse anti-RNA polymerase II CTD repeat YSPTSPS	Abcam	Cat#ab817; RRID: AB_306327
Rat monoclonal anti-BrdU	Abcam	Cat#ab6326; RRID: AB_305426
Mouse monoclonal anti-BrdU	Becton Dickinson	Cat#347580; RRID: AB_10015219
Mouse anti-GAPDH	Abcam	Cat#ab8245; RRID: AB_2107448
Mouse anti-TUBULIN	Sigma-Aldrich	Cat#T6074; RRID: AB_477582
Mouse anti-S9.6	The Francis Crick Institute Cell Services	RRID: AB_2313773

**Chemicals, Peptides, and Recombinant Proteins**		

Adenovirus Ad-Cre-GFP	Vector Biolabs	Cat#1700
Adenovirus Ad-GFP	Vector Biolabs	Cat#1060
Adenovirus Ad-iCre-RFP	Vector Biolabs	Cat#1774
Adenovirus Ad-RFP	Vector Biolabs	Cat#1660
CldU	Sigma-Aldrich	Cat#C6891
IdU	Sigma-Aldrich	Cat#I7125
TMPyP4	Millipore	Cat#613560
Hydroxytamoxifen	Sigma-Aldrich	Cat#H7904
Doxycycline	Sigma-Aldrich	Cat#D9891
Geneticin G418 sulfate	GIBCO	Cat#11811-031
Propidium Iodide	Sigma	Cat#P4864
TelC PNA probe	PANAGENE	Cat#F2003
Proteinase K recombinant	Roche	Cat#40278100
Lipofectamine 2000	Thermo Fisher	Cat#11668027
Fluoroshield with DAPI	Sigma-Aldrich	Cat#F6057

**Critical Commercial Assays**		

RNeasy Mini Kit	QIAGEN	Cat#74106
QIAprep Spin Miniprep Kit	QIAGEN	Cat#27106
Duolink® *In Situ* PLA® Probe Anti-Rabbit MINUS	Sigma-Aldrich	Cat# DUO92005
Duolink® *In Situ* PLA® Probe Anti-Mouse PLUS	Sigma-Aldrich	Cat# DUO92001
Duolink® *In Situ* Detection Reagents Red	Sigma-Aldrich	Cat# DUO92008
Duolink® *In Situ* Wash Buffers, Fluorescence	Sigma-Aldrich	Cat# DUO82049

**Deposited Data**		

Original, unprocessed data	This study	https://doi.org/10.17632/w8zh77nk36.1
Sequencing	This study	GEO: GSE161597

**Experimental Models: Cell Lines**		

Mouse Embryonic Fibroblasts *Rtel1*^*F/F*^	Vannier et al., 2012	N/A
Mouse Embryonic Fibroblasts TamCre *Rtel1*^F/F^	This study	N/A
Mouse Embryonic Fibroblasts Rtel1^*F/F*^;pBabe *Rtel1*^*C1252A/C1255A*^	Sarek et al., 2015	N/A
Mouse Embryonic Fibroblasts *Terf1*^*F/F*^	This study	N/A
Mouse Embryonic Fibroblasts *Rtel1*^F/F^;WT RNaseH1GFP	This study	N/A
Mouse Embryonic Fibroblasts *Rtel1*^F/F^;D210N-RNaseH1GFP	This study	N/A
Mouse Embryonic Fibroblasts *Rtel1*^*+/+*^-V5;WT RNaseH1GFP	This study	N/A
Mouse Embryonic Fibroblasts *Rtel1*^*IA/IA*^-V5;WT-RNaseH1GFP	This study	N/A
Mouse Embryonic Fibroblasts Rtel1^*F/F*^;pBabe *Rtel1*^*C1252A/C1255A*^ ; D210N RNH1-GFP	This study	N/A

**Software and Algorithms**		

Adobe Photoshop CC	Adobe	https://www.adobe.com/es/products/photoshop.html
ImageJ	NIH	https://imagej.nih.gov/ij/
Volocity 6.3	PerkinElmer	https://www.perkinelmer.com/uk/lab-products-and-services/resources/cellular-imaging-software-downloads.html
GraphPad Prism 7	GraphPad	https://www.graphpad.com/
Flow Jo 10	FlowJo	https://www.flowjo.com/
FastQC	Daley and Smith, 2013	https://www.bioinformatics.babraham.ac.uk/projects/fastqc/
nf-core/chipseq	Ewels et al., 2020	https://zenodo.org/record/3966161
picard-tools	Broad Institute	http://broadinstitute.github.io/picard
Pysam	GitHub	https://github.com/pysam-developers/pysam
ggplot2	(Wickham et al., 2016)	https://ggplot2-book.org/
cutadapt	(Martin, 2011)	https://journal.embnet.org/index.php/embnetjournal/article/view/200
Fgsea	(Korotkevich et al., 2019)	https://www.biorxiv.org/content/10.1101/060012v2.full
Trim Galore!	Babraham Bioinformatics	https://www.bioinformatics.babraham.ac.uk/projects/trim_galore/
Nextflow	Tommaso et al., 2017	https://nextflow.io/
Singularity	Kurtzer et al., 2017	https://sylabs.io/guides/2.6/user-guide/quick_start.html
Burrows-Wheeler Alignment tool	Li and Durbin, 2009	http://maq.sourceforge.net
SAMtools	Li et al., 2009	http://samtools.sourceforge.net
BEDTools	Quinlan and Hall, 2010	https://code.google.com/archive/p/bedtools
BamTools	Barnett et al., 2011	https://github.com/pezmaster31/bamtools
bedGraphToBigWig	Kent et al., 2010	http://hgdownload.cse.ucsc.edu/admin/exe/linux.x86_64/
MACS2	Zhang et al., 2008	https://github.com/macs3-project/MACS
HOMER	Heinz et al., 2010	http://homer.ucsd.edu/homer/
featureCounts	Liao et al., 2014	http://subread.sourceforge.net
R	R core team	https://www.r-project.org/
DESeq2	Love et al., 2014	http://www.bioconductor.org/packages/release/bioc/html/DESeq2.html
MultiQC	Ewels et al., 2016	https://multiqc.info/
deepTools	Ramirez et al., 2016	https://deeptools.readthedocs.io/
phantompeakqualtools	Landt et al., 2012	http://www.modencode.org/; https://www.encodeproject.org/
RSEM	Li and Dewey, 2011	http://deweylab.biostat.wisc.edu/rsem
STAR	Dobin et al., 2013	https://code.google.com/archive/p/rna-star
UCSC Table Browser	Karolchik et al., 2004	http://genome.ucsc.edu/
IGV genome browser	Robinson et al., 2011	https://www.broadinstitute.org/igv/
UpSetR	Conway et al., 2017	https://github.com/hms-dbmi/UpSetR/
G4Hunter	Bedrat et al., 2016	http://bioinformatics.ibp.cz.
ChIPseeker	Yu et al., 2015	http://www.bioconductor.org/packages/release/bioc/html/ChIPseeker.html
BioMart	Smedley et al., 2009	http://www.biomart.org
Custom scripts	This study	https://github.com/arpoe/KotsantisP_CellReps_2020

### Resource Availability

#### Lead contact

Further information and requests for reagents should be directed to and will be fulfilled by the Lead Contact, Simon Boulton (simon.boulton@crick.ac.uk).

#### Materials availability

Mouse cell lines generated in this study are available upon request to the Lead Contact (**simon.boulton@crick.ac.uk**).

#### Data and code availability

The accession number for the sequencing data reportedin this paper is GEO: GSE161597. Custom scripts are available at https://github.com/arpoe/KotsantisP_CellReps_2020.
Original data have been deposited at Mendeley Data: https://doi.org/10.17632/w8zh77nk36.1.

### Experimental Model and Subject Details

#### Cell lines and cell culture procedures

Mouse cell lines used in the study are listed in key resource table. MEFs were cultured in Dulbecco’s modified Eagle’s medium (DMEM) supplemented with 10% fetal bovine serum (Invitrogen), L-glutamine, and penicillin-streptomycin. Deletion of floxed alleles in Rtel1f/f and Trf2f/- cells was carried out with either Ad-GFP, Ad-GFP-Cre, Ad-RFP or Ad-RFP-iCre adenovirus (Vector Biolabs). Cells were genotyped by PCR at 96 h post-infection to confirm gene deletion.

To prepare cell lines that inducibly overexpress WT or D210N-RNaseH1GFP, MEFs were infected with M27-WT RNaseH1-GFP-pInducer20 or D210N-RNaseH1-GFP-pInducer20 plasmid and selected with 500 mg ml^-1^ G418 (GIBCO).

Cell lines used were: *Rtel1*^F/F^ MEFs, *Terf1*^*F/F*^ MEFs, Rtel1^*F/F*^;pBabe *Rtel1*^*C1252A/C1255A*^ MEFs, TamCre *Rtel1*^F/F^ MEFs, *Rtel1*^F/F^;WT RNaseH1GFP MEFs, *Rtel1*^F/F^;D210N-RNaseH1GFP MEFs, *Rtel1*^*+/+*^-V5;WT RNaseH1GFP MEFs, *Rtel1*^*IA/IA*^-V5;WT-RNaseH1GFP MEFs and Rtel1^*F/F*^;pBabe *Rtel1*^*C1252A/C1255A*^ ;D210N RNH1-GFP

In order to induce expression of WT RNaseH1-GFP or RNaseH1^D210N^-GFP the relative cell lines were incubated with doxycycline (2 μg/ml) for 48 h.

In order to induce G4 stabilization cells were treated with TMPyP4 (10 μM).

In order to inhibit transcription elongation cells were treated with cordycepin (50 μΜ) for 3.5 h.

### Method Details

#### Expression vectors

In order to prepare M27-WT RNaseH1-GFP-pInducer20 plasmid, human M27-WT RNaseH1-GFP (a kind gift from Dr Kanagaraj Radhakrishnan) was inserted into a pInducer20 lentivirus construct. Catalytically inactive RNaseH1 was created by introducing a D210N point mutation into pINDUCER hM27RNaseH1i-EGFP using the Q5 Site-Directed Mutagenesis kit (NEB # E0554) and the following primers F: TCTGTATACAAACAGTATGTTTAC R: ACCAGTTTATTGATGTTTTG as per the manufacturer’s instructions. All DNA preparations (including PCR clean-up, agarose gel extractions, minipreps, and maxipreps) were done with DNA purification kits from QIAGEN according to the manufacturer’s instructions. All constructs were confirmed by sequencing.

#### Cre-mediated recombination

*Rtel1*^*F/F*^ MEFs were infected with adenovirus expressing the CRE recombinase together with a GFP or RFP marker to inactivate *Rtel1* (Ad-CRE-GFP or Ad-iCre-RFP) or control adenovirus expressing only GFP or RFP (Ad-GFP or Ad-RFP). Samples were processed for analysis 96 ours after infection and loss of RTEL1 was verified by PCR and/or western blot.

#### DNA fiber analysis

Cells were pulse labeled with 25 μM CldU and 250 μM IdU for 15 or 20 min each and harvested. DNA fiber spreads were prepared by spotting 2 μL of cells (5^∗^10^5^ cells per ml in PBS) onto microscope slides followed by lysis with 7 μL of 0.5% SDS, 200 mM Tris-HCl pH 7.4 and 50 mM EDTA. Slides were tilted and DNA spreads fixed in methanol/acetic acid (3:1). HCl-treated fiber spreads were incubated with rat anti-bromodeoxyuridine (detects CldU, abcam, ab6326, 1:1,200) and mouse anti-bromodeoxyuridine (detects IdU, B44, Becton Dickinson, 1:500) for 1 h and incubated with anti-rat IgG AlexaFluor 555 and anti-mouse IgG AlexaFluor 488 (both at 1:500, Molecular Probes) for 1.5 h. Images were acquired using a Zeiss AxioImager M1, equipped with a Hamamatsu digital camera and the Volocity software (Perkin Elmer). Fiber length was analyzed using ImageJ (https://imagej.nih.gov/ij/). For fork speed analysis, during each independent experiment, a minimum of 300 fibers were measured per condition. Fork asymmetry was measured as a percentage of the length ratio of the shortest to the longest fiber of first label origin fibers.

#### Immunofluorescence

For micronuclei analysis, cells grown on coverslips were washed once with PBS and fixed with 4% PFA in PBS for 10 min at room temperature. For all other immunostaining experiments, cells grown on coverslips were washed once with PBS and pre-extracted by incubation in 0.5% Triton X-100 in CSK buffer (10 mM PIPES, 300 mM sucrose, 100 mM NaCl and 3 mM MgCl_2_) for 5 min on ice, washed once with PBS and fixed with 4% PFA in PBS for 10 min at room temperature. For PCNA staining, cells after pre-extraction and fixation were treated with methanol for 10 min at −20°C. Cells were blocked with 3% BSA/10% fetal bovine serum for 1 h at room temperature and incubated overnight with primary antibody diluted in blocking buffer at 4°C. Primary antibodies were mouse anti-phospho-HistoneH2AX (Ser139) (Millipore 05-636, 1:1,000), rabbit anti-53BP1 (Bethyl A300-272A, 1:10,000), mouse RPA32/RP2 (abcam, ab2175, 1:2,000), rabbit pATR-S428 (Cell Signaling, 2853, 1:2,000), mouse PCNA (Santa Cruz, SC-56, 1:3,500), mouse GFP (Roche, 1184460001, 1:1,000), rabbit GFP (abcam, ab290, 1:2,500) and rabbit V5 (Sigma, V8317, 1:3,000). Secondary antibodies were anti-mouse IgG Alexa Fluor 488, anti-mouse IgG Alexa Fluor 488, anti-mouse IgG Alexa Fluor 594, anti-rabbit IgG Alexa Fluor 488 and anti-rabbit IgG Alexa Fluor 594 (all Molecular Probes, 1:500). Coverslips were mounted onto glass slides using Fluoroshield containing 4,6-diamidino-2-phenylindole (DAPI) to counterstain DNA and images acquired as above. Foci analysis was performed with Cell Profiler software (https://cellprofiler.org/). For quantification of nuclear RNaseH1^D210N^-GFP intensity, ImageJ was used to generate nuclear masks based on DAPI staining and mean fluorescence intensities per pixel were quantified per nucleus.

#### Western blotting

Cells were rinsed twice with PBS, lysed in 2x NuPAGE LDS sample buffer (Invitrogen, 13778150) supplemented with 0.1M DTT and sonicated to release DNA-bound proteins. Protein concentration was measured using nanodrop, equal quantities were separated by SDS-PAGE using NuPAGE mini gels (Invitrogen) and transferred onto a nitrocellulose membrane using standard procedures. After transfer, the membrane was blocked in 5% skim milk/ PBST (PBS/ 0.05%Tween-20) for 1 h at room temperature and incubated with the indicated primary antibody (diluted in 5% skim milk/ PBST) overnight at 4°C. The membrane was then washed 5 times for 5 min with PBST, incubated with a horseradish peroxidase-conjugated secondary antibody for 1 h at room temperature, and washed again 5 times for 5 min with PBST. The immunoblot was developed using ECL Western Blotting Reagent (Sigma). All incubations were carried out on a horizontal shaker. Primary antibodies used were mouse GFP (Roche, 1184460001, 1:1,000), rabbit RTEL1 (Novus, 1:3,000), mouse GAPDH (abcam, ab8245), mouse tubulin (Sigma, T6074)

#### Proximity ligation assay

Cells grown on coverslips were pre-extracted in 0.5% NP40 on ice for 4min then washed once with PBS an fixed with 4% formaldehyde in PBS for 15 min at room temperature, washed three times with PBS, blocked with 3% BSA/10% fetal bovine serum and incubated with antibodies mouse RNAPII 8WG16Pol 1:200 and rabbit PCNA 1:200 overnight at 4°C. PLA was performed following the manufacturer’s instructions using the Duolink anti-Mouse MINUS and anti-Rabbit PLUS *In Situ* PLA probes and the Duolink *In Situ* Detection Reagents Red (Olink Bioscience). Images were acquired using a Zeiss AxioImager M1, equipped with a Hamamatsu digital camera and the Volocity software (Perkin Elmer). PLA foci were analyzed using ImageJ (https://imagej.nih.gov/ij/).

#### DRIP QPCR

5x10^6^ cells were collected, washed in PBS and resuspended in 1.6 mL of TRIS-EDTA buffer pH8.0. Cells were lysed by addition 50 μL SDS 20% and 5 μL ProteinaseK 20 mg/ml (Roche), mix gently and incubated overnight at 37°C. DNA was extracted with phenol/chloroform in phase lock tubes, precipitated with EtOH/sodium acetate, washed three times with 70% EtOH, and resuspended in TE. DNA was digested with EcoRI, HindIII, BsrGI, SspI and XbaI (NEB) restriction enzymes overnight at 37°C and DNA was isolated as described above. For DRIP 4.4 μg of digested DNA was diluted in 500 μL TE buffer pH8.0, 50 μL was kept as input and 50 μL of 10x binding buffer (100 mM NPO_4_ pH7.0, 1.4 M NaCl, 0.5% Triton X-100) and 10 μL of S9.6 antibody was added to the rest and incubated overnight at 37°C. Protein A Dynabeads (Roche, 10002D) were added for 2 h. Bound beads were washed 3 times in binding buffer 1x and elution was performed in elution buffer (50 mM Tris pH 8, 10 mM EDTA, 0.5% SDS, Proteinase K) for 45 min at 55°C. DNA was purified as described and resuspended in 10 mM TrisHCl, pH 8.0. Quantitative PCR of immunoprecipitated DNA fragments was performed on Bio-Rad CFX96 Real-Time System C1000 Thermal Cycler using SsoAdvanced Universal SYBR® Green Supermix (Bio-Rad, 1725271).

##### Primers used

Bcl6:

For 5′-CTAATTCTTCCTCTCCTACCCA-3′;
Rev 5′-TTTTTCTCGTGGTGCCTAATACT-3′

B-actin:

For 5′-GAGGGGAGAGGGGGTAAA-3′;
Rev 5′-GAAGCTGTGCTCGCGG-3′

#### PNA FISH

Cells were arrested in colcemid (1 μg/ml) for 4 h and collected by mitotic shock followed by incubation in hypotonic solution (0,075 M KCl) for 20 min at RT. Subsequently cells were washed twice in 3:1 methanol-acetic acid solution and spread on slides. Spreads were incubated for 5 min in 3.6% formaldehyde-0.5% triton-PBS, denatured for 40 min at 72°C in preheated 2xSSC, incubated for 20 min in 0.1 M NaOH, rinsed in water and air-dried. Next, spreads were denatured at 80°C for 2 min with telomere probe (TelC PNA probe) in hybrydisation buffer (10% dextran sulfate, 50% deionised formamide, 2xSSC final) and hybridized for overnight at 37°C in humiditiy chamber. Finally, spreads were washed two times for 20 min in 2xSSC prewarmed to 37°C.

#### IF/TERRA FISH for RNaseA resistant TERRA (in R-loops)

Cells were pre-extracted in 0.5% PBS-Triton, crosslinked for 10 min in 2% PFA-PBS and incubated ON at 37°C with 1 mg/ml RNaseA in PBS to digest RNA not being enaged in R-loops. For IF, cells were blocked for 1 h at RT in blocking buffer (5% milk, 3% BSA, 0.5% Tween, 0.5% NP40 in PBS) followed with 2 h incubation with primary anti-TRF2 antibody (CST #13136S) diluted 1/50 in PBS, 2 washes for 15 min in PBS-400 mM NaCl-0.5% tween-0.5% NP40. Subsequently, cells were incubated with anti-rabbit secondary antibody diluted 1/1000 in PBS (Thermofisher #A32731) for 1 h at RT, followed by 2 washes for 15 min in PBS-400 mM NaCl-0.5% tween-0.5% NP40. Next, cells were crosslinked for 15 min in 2% PFA-PBS, rinsed with PBS, air-dried and incubated ON at 37°C with PNA-TelC probe diluted 1/100 in 10% dextran sulfate-50% formamide-2xSSC. Hybrydisation was followed by 2 washes for 20 min in 50%-formamide-2xSSC and one 20 min wash in 2xSSC.

#### Fluorescence-activated cell sorting (FACS)

For Propidium Iodide (PI)-based determination of DNA content, cells were trypsinised and fixed in 70% ethanol. Cells were then resuspended in an RNase A (20 mg/ml) and propidium iodide (50 mg/ml) solution, passed through a 70 mm cell strainer and the cell cycle distribution of the cells analyzed by flow cytometry, using a 610/20 gate. For EdU/DAPI-based determination of DNA content and new DNA incorporation, cells were incubated with 10 uM EdU for 1 h, trypsinised, and fixed in 2% PFA for 10 mins. Newly incorporated DNA was stained using a Click-iT EdU Alexa Fluor 647 kit and DNA was stained using DAPI. The cell cycle distribution of the cells was analyzed by flow cytometry, using 440/40 and 610/20 gates to identify DNA content (DAPI) and newly synthesized DNA (Alexa-647) respectively. Gating and analysis was performed manually using FlowJo v10 (FlowJo).

#### DRIP-seq

5x10^6^ cells were collected, washed in PBS and resuspended in 1.6 mL of TRIS-EDTA buffer pH8.0. Cells were lysed by addition 50 μL SDS 20% and 5 μL ProteinaseK 20 mg/ml (Roche), mix gently and incubated overnight at 37°C. DNA was extracted with phenol/chloroform in phase lock tubes, precipitated with EtOH/sodium acetate, washed three times with 70% EtOH, and resuspended in TE. DNA was digested with EcoRI, HindIII, BsrGI, SspI and XbaI (NEB) restriction enzymes overnight at 37°C and DNA was isolated as described above and 4.4 μg of digested DNA was diluted in 500 μL TE buffer pH8.0 aliquots. For DRIP-seq, three IPs with the S9.6 antibody per condition were performed in parallel to obtain enough material for library construction. For each aliquot of digested DNA, 50 μL was kept as input and 50 μL of 10x binding buffer (100 mM NPO_4_ pH7.0, 1.4 M NaCl, 0.5% Triton X-100) and 10 μL of S9.6 antibody was added to the rest and incubated overnight at 37°C. Protein A Dynabeads (Roche 10002D) were added for 2 h. Bound beads were washed 3 times in binding buffer 1x and elution was performed in elution buffer (50 mM Tris pH 8, 10 mM EDTA, 0.5% SDS, Proteinase K) for 45 min at 55°C. DNA was purified as described and resuspended in 20 μL 10 mM TrisHCl, pH 8.0. Three IPs per condition were pooled and 5 μL was withdrawed and efficiency of IP was assessed by QPCR (see above). Once quality of S9.6 IP was verified, pooled IPs were treated with RNaseA for 1 h at 37°C and DNA was fragmented using a Covaris LE220 ultrasonicator with the following settings: iterations: 25, duration: 10 s, peak powerL 450, duty factor: 25%, cycles/bursts; 200. Following fragmentation, DNA was converted into illumina compatible libraries using the NEBNext Ultra II kit according to manufacturer’s instructions. Libraries were then sequenced on an Illumina HiSeq 4000 using single ended 100 bp reads.

#### RNA-seq

RNA samples were quantified using the Agilent BioAnalyser, and libraries were prepared using the KAPA mRNA HyperPrep kit according to the manufacturer’s instructions with an input of 1 μg RNA. The libraries were pooled to 4 nM and sequenced on the HiSeq 4000 with 75 bp single ended reads

#### Sequencing read alignment

Adaptor trimming was performed with cutadapt (version 1.9.1) (Martin, 2011) with parameters “–minimum-length=25–quality-cutoff=20 -a AGATCGGAAGAGC -A AGATCGGAAGAGC.”

The RSEM package (version 1.3.0) (Li and Dewey, 2011) in conjunction with the STAR alignment algorithm (version 2.5.2a) (Dobin et al., 2013) was used for the mapping and subsequent gene-level counting of the sequenced reads with respect to mm10 RefSeq genes downloaded from the UCSC Table Browser (Karolchik et al., 2004) on 19th February 2016. The parameters used were “–star-output-genome-bam–forward-prob 0–paired-end.”

#### Differential gene expression analysis

Differential expression analysis was performed with the DESeq2 package (version 1.28.1) (Love et al., 2014) within the R programming environment (version 4.0.0.) (http://www.R-project.org/). An adjusted p value of ≤ 0.01 was used as the significance threshold for the identification of differentially expressed genes.

For visualization of Terf1 dependent gene expression changes, Terf1 itself was excluded from the visualization.

Heatmaps to show differentially expressed genes were generated on count data that were normalized and norm transformed using the DESeq2 package, and depicted using “pheatmap” with scaling by row.

Venn diagrams were generated using the R environment and https://www.meta-chart.com/venn for visualization.

#### Definition of G-quadruplex regulated promoters

G-quadruplex structures were predicted using the “G4Hunter” package (Bedrat et al., 2016) using a threshold at a score of 1.5. G-quadruplex structures were assigned to promoters strand specifically with a threshold of 1kb from the transcriptional start site using the “ChIPseeker” package (v. 1.24.0) (Yu et al., 2015).

#### DRIP-Seq analysis

The nf-core/chipseq pipeline (version 1.2.1; (Ewels et al., 2020); https://zenodo.org/record/3966161) written in the Nextflow domain specific language (version 19.10.0; (Tommaso et al., 2017)) was used to perform the primary analysis of the samples in conjunction with Singularity (version 2.6.0; (Kurtzer et al., 2017)). The command used was “nextflow run nf-core/chipseq–input design.csv–genome mm10–gtf refseq_genes.gtf–single_end–narrow_peak -profile crick -r 1.2.1.” To summarize, the pipeline performs adaptor trimming (Trim Galore! - https://www.bioinformatics.babraham.ac.uk/projects/trim_galore/), read alignment (BWA - (Li and Durbin, 2009)) and filtering (SAMtools - (Li et al., 2009); BEDTools - (Quinlan and Hall, 2010); BamTools - (Barnett et al., 2011); pysam - https://github.com/pysam-developers/pysam; picard-tools - http://broadinstitute.github.io/picard), normalized coverage track generation (BEDTools - (Quinlan and Hall, 2010); bedGraphToBigWig - (Kent et al., 2010)), peak calling (MACS2 - (Zhang et al., 2008)) and annotation relative to gene features (HOMER - (Heinz et al., 2010)), consensus peak set creation (BEDTools -(Quinlan and Hall, 2010)), differential binding analysis (featureCounts - (Liao et al., 2014); R - R Core Team; DESeq2 - (Love et al., 2014)) and extensive QC and version reporting (MultiQC - (Ewels et al., 2016); FastQC - https://www.bioinformatics.babraham.ac.uk/projects/fastqc/; preseq - (Daley and Smith, 2013); deepTools - (Ramírez et al., 2016); phantompeakqualtools - (Landt et al., 2012)). Tracks illustrating read coverage and representative peaks were visualized using the IGV genome browser (Robinson et al., 2011). Further analysis was performed in the R programming environment (version 4.0.0.). Only the peaks were considered that have a score in the top decile per sample. Peaks were annotated to genes using the UCSC annotation with mm10. A peak was considered to be in a promoter, if within ± 1 kb of the transcriptional start site. Promoters and genes with R-loop peaks were compared between different samples by determining overlaps, visualized using the “UpSetR” package (v. 1.4.0) (Conway et al., 2017).

#### Enrichment analysis for genes associated with fragile sites and R-loops

Early replicating fragile sites were obtained from GSE43504 (Barlow et al., 2013). Common fragile sites were obtained from the HumCFS database (Kumar et al., 2019) and converted to the mouse orthologs using BioMart (Smedley et al., 2009). Enrichment analysis for fragile sites and R-loops was performed using the “fgsea” package (v. 1.14.0) (Korotkevich et al., 2019) and visualized with a custom script based on the “ggplot2” package (v. 3.3.0) (Wickham et al., 2016).

### Quantification and Statistical Analysis

Statistical significance of non-sequencing experiments was determined with the tests stated in the figure legends using GraphPad PRISM software. All data are from a minimum of two independent experiments. Specific biological replicate numbers (n) for each experiment can be found in the corresponding figure legends. Statistical analysis of RNA-seq was performed using the DESeq2 framework in R. Statistical tests are performed with negative binomial testing. Statistical analysis of gene expression and GSEA datasets was performed using the fgsea framework in R with the associated t-based testing statistics. Statistical analysis of DRIP-seq was performed using peak calling with MACS2 and the associated testing statistics. Gene set enrichment for DRIP-seq peaks was assessed with the fgsea framework in R as detailed in the methods section. Statistically significant differences are labeled with one, two, three or four asterisks if p < 0.05, p < 0.01, p < 0.001 or p < 0.0001, respectively.

## Supplemental Information

Document S1. Figures S1–S5

Document S2. Article plus Supplemental Information

## References

[bib1] Arora R., Lee Y., Wischnewski H., Brun C.M., Schwarz T., Azzalin C.M. (2014). RNaseH1 regulates TERRA-telomeric DNA hybrids and telomere maintenance in ALT tumour cells. Nat. Commun..

[bib2] Barber L.J., Youds J.L., Ward J.D., McIlwraith M.J., O’Neil N.J., Petalcorin M.I., Martin J.S., Collis S.J., Cantor S.B., Auclair M. (2008). RTEL1 maintains genomic stability by suppressing homologous recombination. Cell.

[bib3] Barlow J.H., Faryabi R.B., Callén E., Wong N., Malhowski A., Chen H.T., Gutierrez-Cruz G., Sun H.W., McKinnon P., Wright G. (2013). Identification of early replicating fragile sites that contribute to genome instability. Cell.

[bib4] Barnett D.W., Garrison E.K., Quinlan A.R., Strömberg M.P., Marth G.T. (2011). BamTools: a C++ API and toolkit for analyzing and managing BAM files. Bioinformatics.

[bib5] Bedrat A., Lacroix L., Mergny J.L. (2016). Re-evaluation of G-quadruplex propensity with G4Hunter. Nucleic Acids Res..

[bib6] Bellelli R., Youds J., Borel V., Svendsen J., Pavicic-Kaltenbrunner V., Boulton S.J. (2020). Synthetic Lethality between DNA Polymerase Epsilon and RTEL1 in Metazoan DNA Replication. Cell Rep..

[bib7] Björkman A., Johansen S.L., Lin L., Schertzer M., Kanellis D.C., Katsori A.M., Christensen S.T., Luo Y., Andersen J.S., Elsässer S.J. (2020). Human RTEL1 associates with Poldip3 to facilitate responses to replication stress and R-loop resolution. Genes Dev..

[bib8] Chappidi N., Nascakova Z., Boleslavska B., Zellweger R., Isik E., Andrs M., Menon S., Dobrovolna J., Balbo Pogliano C., Matos J. (2020). Fork Cleavage-Religation Cycle and Active Transcription Mediate Replication Restart after Fork Stalling at Co-transcriptional R-Loops. Mol. Cell.

[bib9] Chen J.Y., Zhang X., Fu X.D., Chen L. (2019). R-ChIP for genome-wide mapping of R-loops by using catalytically inactive RNASEH1. Nat. Protoc..

[bib10] Conway J.R., Lex A., Gehlenborg N. (2017). UpSetR: an R package for the visualization of intersecting sets and their properties. Bioinformatics.

[bib11] Daley T., Smith A.D. (2013). Predicting the molecular complexity of sequencing libraries. Nat. Methods.

[bib12] De Magis A., Manzo S.G., Russo M., Marinello J., Morigi R., Sordet O., Capranico G. (2019). DNA damage and genome instability by G-quadruplex ligands are mediated by R loops in human cancer cells. Proc. Natl. Acad. Sci. USA.

[bib13] Ding H., Schertzer M., Wu X., Gertsenstein M., Selig S., Kammori M., Pourvali R., Poon S., Vulto I., Chavez E. (2004). Regulation of murine telomere length by Rtel: an essential gene encoding a helicase-like protein. Cell.

[bib14] Dobin A., Davis C.A., Schlesinger F., Drenkow J., Zaleski C., Jha S., Batut P., Chaisson M., Gingeras T.R. (2013). STAR: ultrafast universal RNA-seq aligner. Bioinformatics.

[bib15] Duquette M.L., Handa P., Vincent J.A., Taylor A.F., Maizels N. (2004). Intracellular transcription of G-rich DNAs induces formation of G-loops, novel structures containing G4 DNA. Genes Dev..

[bib16] Ewels P., Magnusson M., Lundin S., Käller M. (2016). MultiQC: summarize analysis results for multiple tools and samples in a single report. Bioinformatics.

[bib17] Ewels P.A., Peltzer A., Fillinger S., Patel H., Alneberg J., Wilm A., Garcia M.U., Di Tommaso P., Nahnsen S. (2020). The nf-core framework for community-curated bioinformatics pipelines. Nat. Biotechnol..

[bib18] Ginno P.A., Lott P.L., Christensen H.C., Korf I., Chédin F. (2012). R-loop formation is a distinctive characteristic of unmethylated human CpG island promoters. Mol. Cell.

[bib19] Glover T.W., Wilson T.E., Arlt M.F. (2017). Fragile sites in cancer: more than meets the eye. Nat. Rev. Cancer.

[bib20] Hamperl S., Bocek M.J., Saldivar J.C., Swigut T., Cimprich K.A. (2017). Transcription-Replication Conflict Orientation Modulates R-Loop Levels and Activates Distinct DNA Damage Responses. Cell.

[bib21] Heinz S., Benner C., Spann N., Bertolino E., Lin Y.C., Laslo P., Cheng J.X., Murre C., Singh H., Glass C.K. (2010). Simple combinations of lineage-determining transcription factors prime cis-regulatory elements required for macrophage and B cell identities. Mol. Cell.

[bib22] Helmrich A., Ballarino M., Tora L. (2011). Collisions between replication and transcription complexes cause common fragile site instability at the longest human genes. Mol. Cell.

[bib23] Karolchik D., Hinrichs A.S., Furey T.S., Roskin K.M., Sugnet C.W., Haussler D., Kent W.J. (2004). The UCSC Table Browser data retrieval tool. Nucleic Acids Res..

[bib24] Kent W.J., Zweig A.S., Barber G., Hinrichs A.S., Karolchik D. (2010). BigWig and BigBed: enabling browsing of large distributed datasets. Bioinformatics.

[bib62] Korotkevich. 2019. https://www.biorxiv.org/content/10.1101/060012v2.full.

[bib25] Kumar R., Nagpal G., Kumar V., Usmani S.S., Agrawal P., Raghava G.P.S. (2019). HumCFS: a database of fragile sites in human chromosomes. BMC Genomics.

[bib26] Kurtzer G.M., Sochat V., Bauer M.W. (2017). Singularity: Scientific containers for mobility of compute. PLoS ONE.

[bib27] Landt S.G., Marinov G.K., Kundaje A., Kheradpour P., Pauli F., Batzoglou S., Bernstein B.E., Bickel P., Brown J.B., Cayting P. (2012). ChIP-seq guidelines and practices of the ENCODE and modENCODE consortia. Genome Res..

[bib28] Li B., Dewey C.N. (2011). RSEM: accurate transcript quantification from RNA-Seq data with or without a reference genome. BMC Bioinformatics.

[bib29] Li H., Durbin R. (2009). Fast and accurate short read alignment with Burrows-Wheeler transform. Bioinformatics.

[bib30] Li H., Handsaker B., Wysoker A., Fennell T., Ruan J., Homer N., Marth G., Abecasis G., Durbin R., 1000 Genome Project Data Processing Subgroup (2009). The Sequence Alignment/Map format and SAMtools. Bioinformatics.

[bib31] Liao Y., Smyth G.K., Shi W. (2014). featureCounts: an efficient general purpose program for assigning sequence reads to genomic features. Bioinformatics.

[bib32] Love M.I., Huber W., Anders S. (2014). Moderated estimation of fold change and dispersion for RNA-seq data with DESeq2. Genome Biol..

[bib60] Martin M. (2011). Cutadapt removes adapter sequences from high-throughput sequencing reads. EMBnet.journal.

[bib33] Nguyen D.T., Voon H.P.J., Xella B., Scott C., Clynes D., Babbs C., Ayyub H., Kerry J., Sharpe J.A., Sloane-Stanley J.A. (2017). The chromatin remodelling factor ATRX suppresses R-loops in transcribed telomeric repeats. EMBO Rep..

[bib34] Nguyen H.D., Yadav T., Giri S., Saez B., Graubert T.A., Zou L. (2017). Functions of Replication Protein A as a Sensor of R Loops and a Regulator of RNaseH1. Mol. Cell.

[bib35] Papadopoulou C., Guilbaud G., Schiavone D., Sale J.E. (2015). Nucleotide Pool Depletion Induces G-Quadruplex-Dependent Perturbation of Gene Expression. Cell Rep..

[bib36] Quinlan A.R., Hall I.M. (2010). BEDTools: a flexible suite of utilities for comparing genomic features. Bioinformatics.

[bib37] Ramírez F., Ryan D.P., Grüning B., Bhardwaj V., Kilpert F., Richter A.S., Heyne S., Dündar F., Manke T. (2016). deepTools2: a next generation web server for deep-sequencing data analysis. Nucleic Acids Res..

[bib38] Robinson J.T., Thorvaldsdóttir H., Winckler W., Guttman M., Lander E.S., Getz G., Mesirov J.P. (2011). Integrative genomics viewer. Nat. Biotechnol..

[bib39] Sarek G., Vannier J.B., Panier S., Petrini J.H.J., Boulton S.J. (2015). TRF2 recruits RTEL1 to telomeres in S phase to promote t-loop unwinding. Mol. Cell.

[bib40] Sarek G., Kotsantis P., Ruis P., Van Ly D., Margalef P., Borel V., Zheng X.F., Flynn H.R., Snijders A.P., Chowdhury D. (2019). CDK phosphorylation of TRF2 controls t-loop dynamics during the cell cycle. Nature.

[bib41] Sarkies P., Reams C., Simpson L.J., Sale J.E. (2010). Epigenetic instability due to defective replication of structured DNA. Mol. Cell.

[bib42] Sarkies P., Murat P., Phillips L.G., Patel K.J., Balasubramanian S., Sale J.E. (2012). FANCJ coordinates two pathways that maintain epigenetic stability at G-quadruplex DNA. Nucleic Acids Res..

[bib43] Schiavone D., Guilbaud G., Murat P., Papadopoulou C., Sarkies P., Prioleau M.N., Balasubramanian S., Sale J.E. (2014). Determinants of G quadruplex-induced epigenetic instability in REV1-deficient cells. EMBO J..

[bib44] Schiavone D., Jozwiakowski S.K., Romanello M., Guilbaud G., Guilliam T.A., Bailey L.J., Sale J.E., Doherty A.J. (2016). PrimPol Is Required for Replicative Tolerance of G Quadruplexes in Vertebrate Cells. Mol. Cell.

[bib45] Sfeir A., Kosiyatrakul S.T., Hockemeyer D., MacRae S.L., Karlseder J., Schildkraut C.L., de Lange T. (2009). Mammalian telomeres resemble fragile sites and require TRF1 for efficient replication. Cell.

[bib46] Skourti-Stathaki K., Kamieniarz-Gdula K., Proudfoot N.J. (2014). R-loops induce repressive chromatin marks over mammalian gene terminators. Nature.

[bib47] Skourti-Stathaki K., Torlai Triglia E., Warburton M., Voigt P., Bird A., Pombo A. (2019). R-Loops Enhance Polycomb Repression at a Subset of Developmental Regulator Genes. Mol. Cell.

[bib48] Smedley D., Haider S., Ballester B., Holland R., London D., Thorisson G., Kasprzyk A. (2009). BioMart--biological queries made easy. BMC Genomics.

[bib49] Takedachi A., Despras E., Scaglione S., Guérois R., Guervilly J.H., Blin M., Audebert S., Camoin L., Hasanova Z., Schertzer M. (2020). SLX4 interacts with RTEL1 to prevent transcription-mediated DNA replication perturbations. Nat. Struct. Mol. Biol..

[bib50] Tommaso P.D., Floden E.W., Magis C., Palumbo E., Notredame C. (2017). [Nextflow, an efficient tool to improve computation numerical stability in genomic analysis]. Biol. Aujourdhui.

[bib51] Vannier J.B., Pavicic-Kaltenbrunner V., Petalcorin M.I., Ding H., Boulton S.J. (2012). RTEL1 dismantles T loops and counteracts telomeric G4-DNA to maintain telomere integrity. Cell.

[bib52] Vannier J.B., Sandhu S., Petalcorin M.I., Wu X., Nabi Z., Ding H., Boulton S.J. (2013). RTEL1 is a replisome-associated helicase that promotes telomere and genome-wide replication. Science.

[bib53] Vannier J.B., Sarek G., Boulton S.J. (2014). RTEL1: functions of a disease-associated helicase. Trends Cell Biol..

[bib54] Varshney D., Spiegel J., Zyner K., Tannahill D., Balasubramanian S. (2020). The regulation and functions of DNA and RNA G-quadruplexes. Nat. Rev. Mol. Cell Biol..

[bib59] Wickham H., Navarro D., Pedersen T., Lin (2016). ggplot2: elegant graphics for data analysi.

[bib55] Wu W., Bhowmick R., Vogel I., Özer Ö., Ghisays F., Thakur R.S., Sanchez de Leon E., Richter P.H., Ren L., Petrini J.H. (2020). RTEL1 suppresses G-quadruplex-associated R-loops at difficult-to-replicate loci in the human genome. Nat. Struct. Mol. Biol..

[bib56] Yadav P., Owiti N., Kim N. (2016). The role of topoisomerase I in suppressing genome instability associated with a highly transcribed guanine-rich sequence is not restricted to preventing RNA:DNA hybrid accumulation. Nucleic Acids Res..

[bib57] Yu G., Wang L.G., He Q.Y. (2015). ChIPseeker: an R/Bioconductor package for ChIP peak annotation, comparison and visualization. Bioinformatics.

[bib58] Zhang Y., Liu T., Meyer C.A., Eeckhoute J., Johnson D.S., Bernstein B.E., Nusbaum C., Myers R.M., Brown M., Li W., Liu X.S. (2008). Model-based analysis of ChIP-Seq (MACS). Genome Biol..

