# Supplementary material for: RTEL1 Regulates G4/R-Loops to Avert Replication-Transcription Collisions
Source: Cell Rep. 2020 Dec 22;33(12):108546. doi: 10.1016/j.celrep.2020.108546 (PMC7773548; doi:10.1016/j.celrep.2020.108546)
Supplement: Document S1. Figures S1–S5 [file mmc1.pdf]

Cell Reports, Volume *33*

Supplemental Information

RTEL1 Regulates G4/R-Loops to Avert Replication-Transcription Collisions

Panagiotis Kotsantis, Sandra Segura-Bayona, Pol Margalef, Paulina Marzec, Phil Ruis, Graeme Hewitt, Roberto Bellelli, Harshil Patel, Robert Goldstone, Anna R. Poetsch, and Simon J. Boulton

Supplementary Figure 1

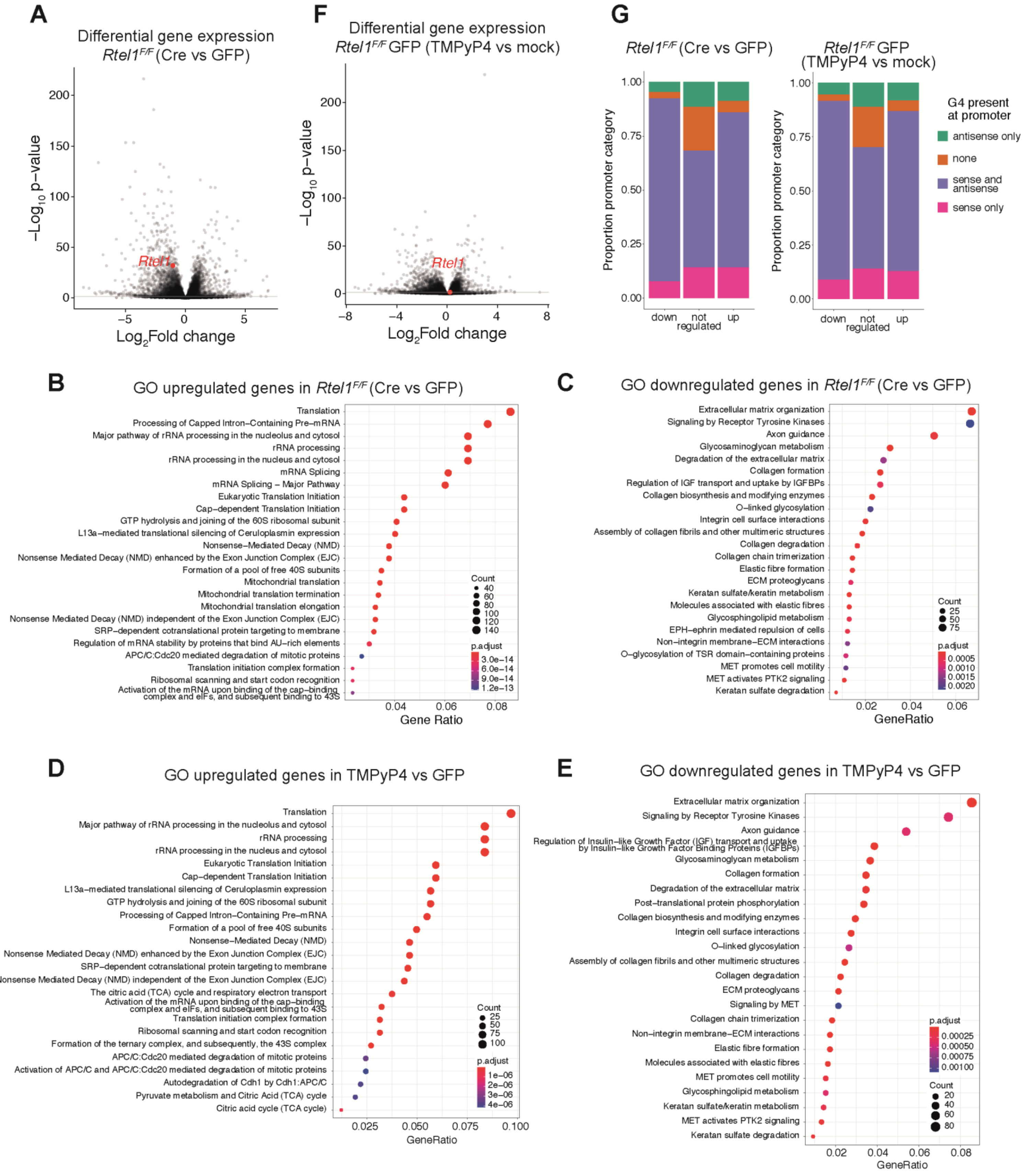

Supplementary Figure 1. Gene expression and gene ontology analysis of *Rtel1* loss and G4 stabilisation. Related to Figure 1.

(A) *Rtel1*$^{F/F}$ MEFs were infected with GFP or Cre-GFP adenovirus and collected after 96 h. RNA was isolated and gene expression levels were analysed. Scatterplot of differential gene expression measures highlighting *Rtel1* in red and the significance threshold of $p < 0.01$ in grey.

(B) Gene Ontology (GO) term analysis of enriched pathways among the upregulated genes upon *Rtel1* deletion.

(C) GO term analysis of enriched pathways among the downregulated genes upon *Rtel1* deletion.

(D) GO term analysis of enriched pathways among the upregulated genes upon TMyP4 treatment.

(E) GO term analysis of enriched pathways among the downregulated genes upon TMyP4 treatment.

(F) *Rtel1*$^{F/F}$ MEFs were infected with GFP or treated with TMPyP4 and collected after 48 h. RNA was isolated and gene expression levels were analysed. Scatterplot of differential gene expression measures highlighting *Rtel1* in red and the significance threshold of $p < 0.01$ in grey.

(G) Overlaps of promoters (transcriptional start site +/- 1 kb) of differentially expressed genes with potential G4-DNA forming sequences of samples that *Rtel1* was deleted (*Rtel1*$^{F/F}$, Cre vs GFP) or treated with TMPyP4 (*Rtel1*$^{F/F}$ GFP, TMPyP4 vs mock).

Supplementary Figure 2

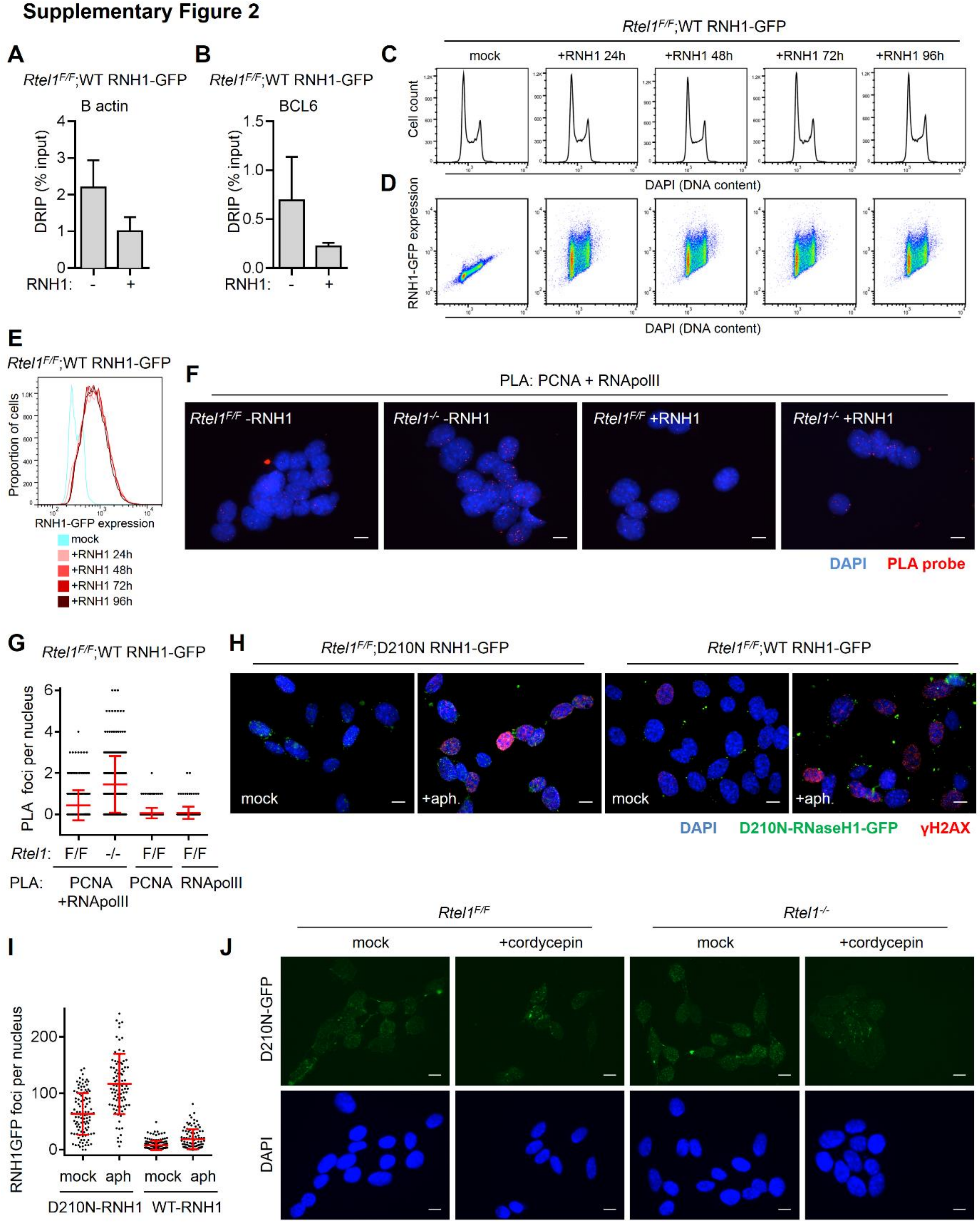

Supplementary Figure 2. Characterisation of *Rtel1$^{F/F}$*;WT RNH1-GFP MEFs. Related to Figure 2.

(A) *Rtel1$^{F/F}$*;WT RNH1-GFP MEFs were treated with doxycycline for 48 h, cells were collected, genomic DNA was isolated, R-loops were isolated by DRIP and QPCR was performed for B actin.

(B) *Rtel1$^{F/F}$*;WT RNH1-GFP MEFs were treated with doxycycline for 48 h, cells were collected, genomic DNA was isolated, R-loops were isolated by DRIP and QPCR was performed for BCL6.

(C) *Rtel1$^{F/F}$*;WT RNH1-GFP MEFs were treated with doxycycline for 24, 48, 72 and 96 h, collected, fixed, stained with propidium iodide and cell cycle profile was analysed by FACS. (≥10,000 cells/condition, n=3).

(D) *Rtel1$^{F/F}$*;WT RNH1-GFP MEFs were treated with doxycycline for 24, 48, 72 and 96 h, collected, fixed and GFP expression was analysed by FACS. (≥10,000 cells/condition, n=3)

(E) Quantification of (D).

(F) Representative images of PLA of Figure 3C.

(G) *Rtel1$^{F/F}$*;WT RNH1-GFP MEFs were infected with GFP or Cre-GFP adenovirus, after 96 h cells were fixed and PLA was performed with the indicated antibodies. (n=1)

(H) *Rtel1$^{F/F}$*;WT RNH1-GFP MEFs were treated with doxycycline for 48 h, incubated with aphidicolin (0.2 μM) for 6 h, pre-extracted, fixed and immunostained for GFP and γH2AX. Representative images of RNaseH1^{D210N}-GFP (green) and γH2AX (red) immunostaining.

(I) Quantification of D210N- and WT- RNaseH1-GFP foci per nucleus of (H). Data are represented as mean ± SD (n=1).

(J) Representative images of RNaseH1^{D210N}-GFP (green) immunostaining of Figure 3F.

Supplementary Figure 3

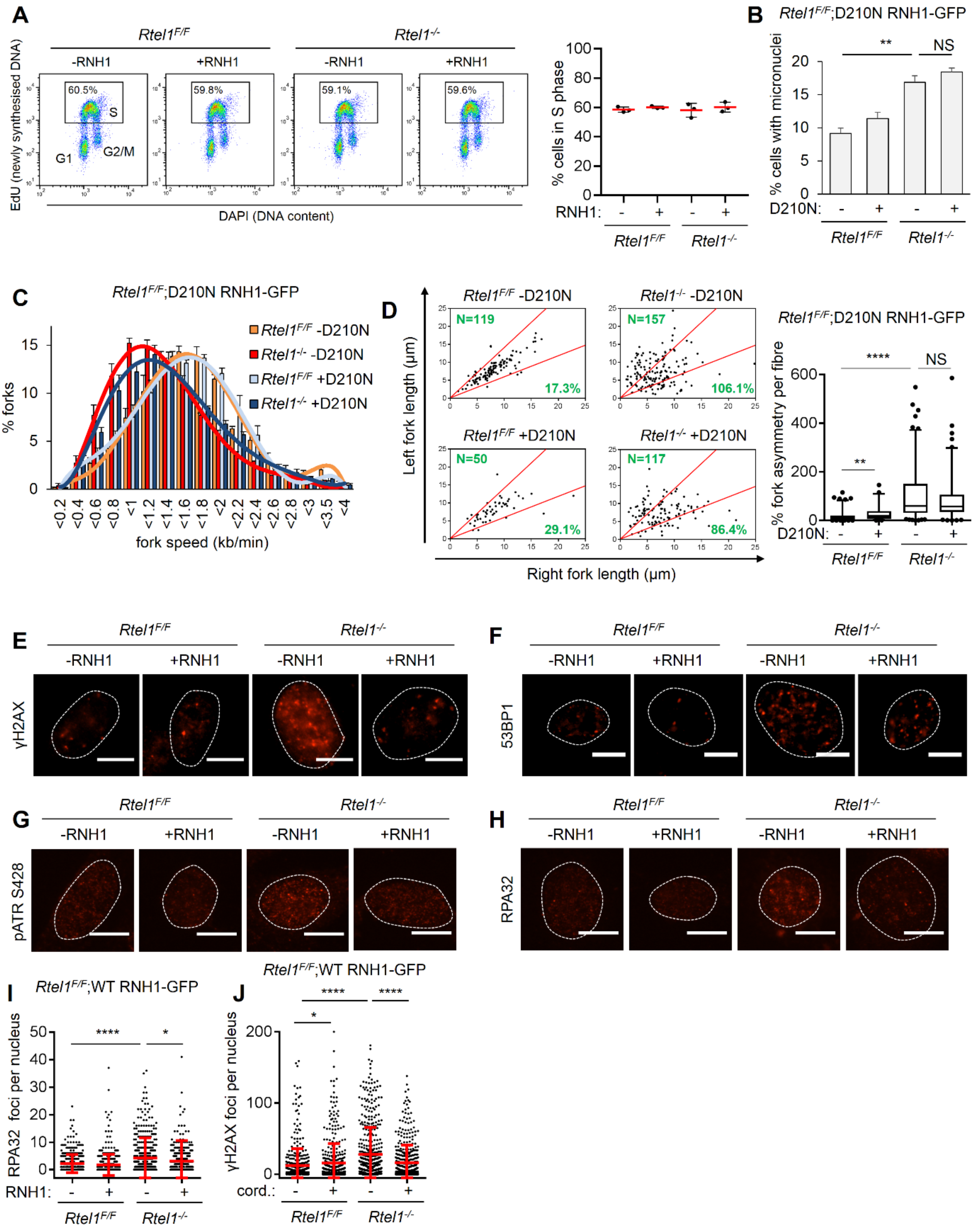

Supplementary Figure 3. RNaseH1^{D210N}-GFP overexpression does not rescue *Rtel1* loss-induced replication stress Related to Figure 3.

(A) *Rtel1$^{F/F}$*;WT RNH1-GFP MEFs were treated as in Figure 3A, incubated with EdU, collected, fixed, stained with propidium iodide and newly incorporated DNA (indicating S-phase) was analysed by FACS. Left panel: Representative images of FACS analysis. Right panel: Quantification of cells in S phase. Data are represented as mean ± SD (n=3).

(B) *Rtel1$^{F/F}$*;D210N RNH1-GFP MEFs were infected with RFP or iCre-RFP adenovirus, after 48 h doxycycline was added and cells were collected after 48 h, fixed and percentage of cells with micronuclei was quantified. Data are represented as mean ± SE (n=3).

(C) *Rtel1$^{F/F}$*;D210N RNH1-GFP MEFs were treated as in (B) and DNA fibre assay was performed. Distribution of replication fork speeds of DNA fibres. Data are represented as mean ± SE (n=3).

(D) Left panel: Scatter plot of fork asymmetry of DNA fibres prepared as in (B). Right panel: Quantification of fork asymmetry of DNA fibres prepared as in (C). In box plots, horizontal line denotes the mean; whiskers denote the 5th and 95th percentiles. (n=3).

(E) Representative images of γH2AX immunostaining of Figure 4E.

(F) Representative images of 53BP1 immunostaining of Figure 4F.

(G) Representative images of pATR-S428 immunostaining of Figure 4G.

(H) *Rtel1$^{F/F}$*;WT RNH1-GFP MEFs were infected with GFP or Cre-GFP adenovirus, after 48 h doxycycline was added and cells were collected after 48 h, fixed and immunostained for RPA32. Representative images of RPA32 immunostaining.

(I) Quantification of number of RPA32 foci per nucleus as prepared in (H). Data are represented as mean ± SD (n=2).

(J) *Rtel1$^{F/F}$*;D210N RNH1-GFP MEFs were infected with RFP or iCre-RFP adenovirus, after 96 h treated with cordycepin (50 μM) for 3.5 h, pre-extracted, fixed and immunostained for γH2AX. Quantification of γH2AX foci per nucleus of cells. Data are represented as mean ± SD (n=3).

P values determined by unpaired T-test *p<0.05, **p<0.01, ****p<0.0001. Scale bars: 10 μm.

Supplementary Figure 4

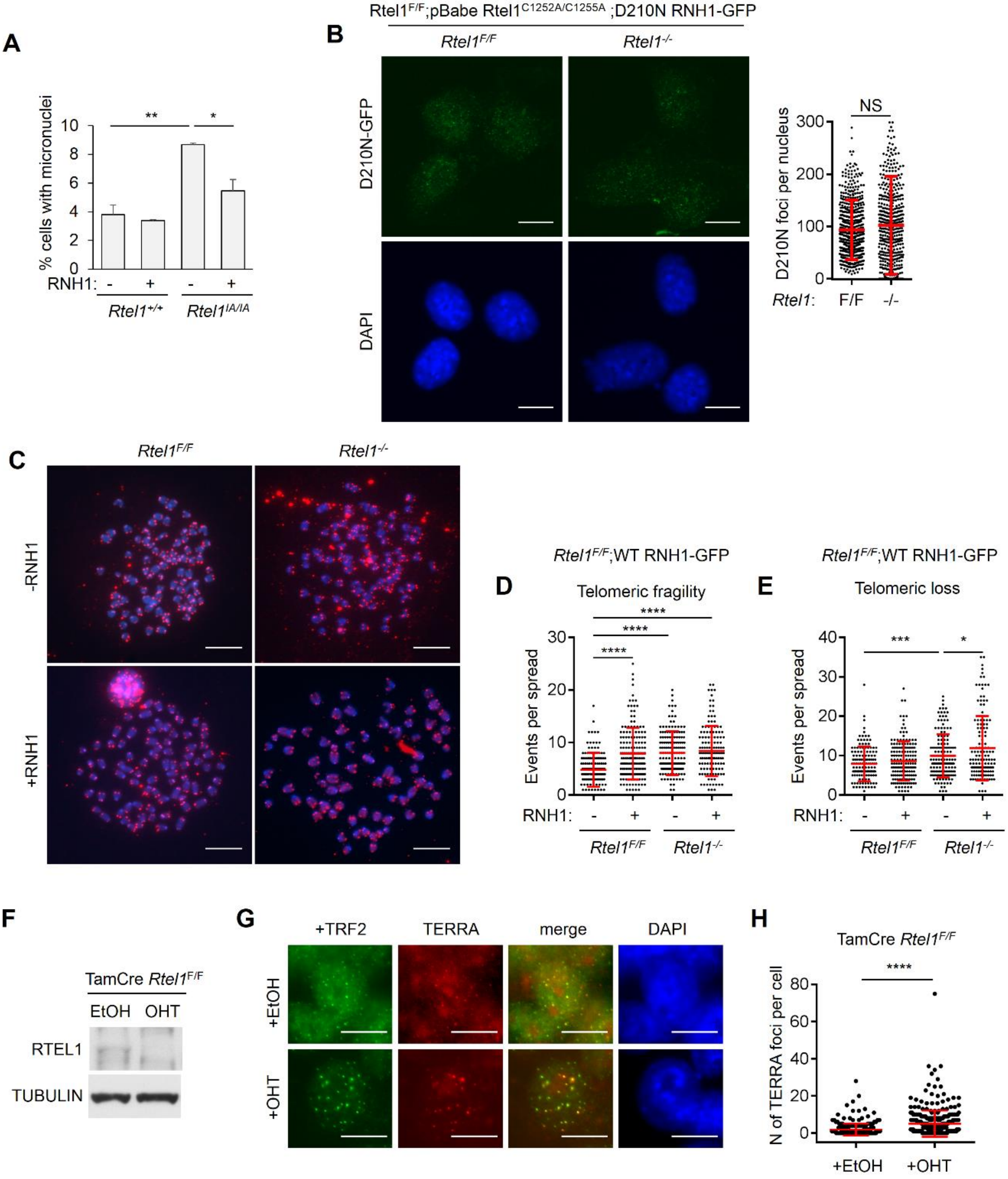

Supplementary Figure 4. Overexpression of WT RNaseH1-GFP does not rescue *Rtel1* loss-induced telomeric stress Related to Figure 3.

(A) *Rtel1*$^{+/+}$-V5;WT RNH1-GFP and *Rtel1*$^{IA/IA}$-V5;WT RNH1-GFP MEFs were treated with doxycycline for 48 h, fixed and percentage of cells with micronuclei was quantified. Data are represented as mean ± SE (n=3).

(B) *Rtel1*$^{F/F}$ *;pBabe Rtel1*$^{C1252A/C1255A}$;D210N RNH1-GFP MEFs were infected with RFP or iCre-RFP adenovirus, after 48 h doxycycline was added and after 48 h, cells were pre-extracted, fixed and immunostained for GFP. Left panel: Representative images of RNaseH1^{D210N}-GFP. Right panel: Quantification of RNaseH1^{D210N}-GFP foci per nucleus of cells. Data are represented as mean ± SD (n=4).

(C) *Rtel1*$^{F/F}$;WT RNH1-GFP MEFs were infected with GFP or Cre-GFP adenovirus, after 48 h doxycycline was added and cells were collected after 48 h, metaphase spreads were prepared and telomeric FISH was performed. Representative images of metaphase spreads of each condition.

(D) Quantification of telomeric fragility in telomeric FISH experiment as performed in (C). Data are represented as mean ± SD (n=4).

(E) Quantification of telomeric loss in telomeric FISH experiment as performed in (C). Data are represented as mean ± SD (n=4).

(F) TamCre *Rtel1*$^{F/F}$ MEFs were incubated with EtOH or OHT (0.5 μM) for 96 h, cells were collected, lysed, whole cell extracts were analysed by SDS-PAGE and immunoblotted for RTEL1 and TUBULIN.

(G) *Rtel1*$^{F/F}$ MEFs were treated as in (F), cells were collected metaphase spreads prepared and IF/FISH for TRF2 and TERRA was performed. Representative images of TERRA and TRF2 IF/FISH.

(H) Quantification of number of TERRA foci per nucleus of cells treated as in (F). Data are represented as mean ± SD (n=2).

P values determined by unpaired T-test *p<0.05, **p<0.01, ***p<0.001, ****p<0.0001 NS: non-significant. Scale bars: 10 μm.

Supplementary Figure 5

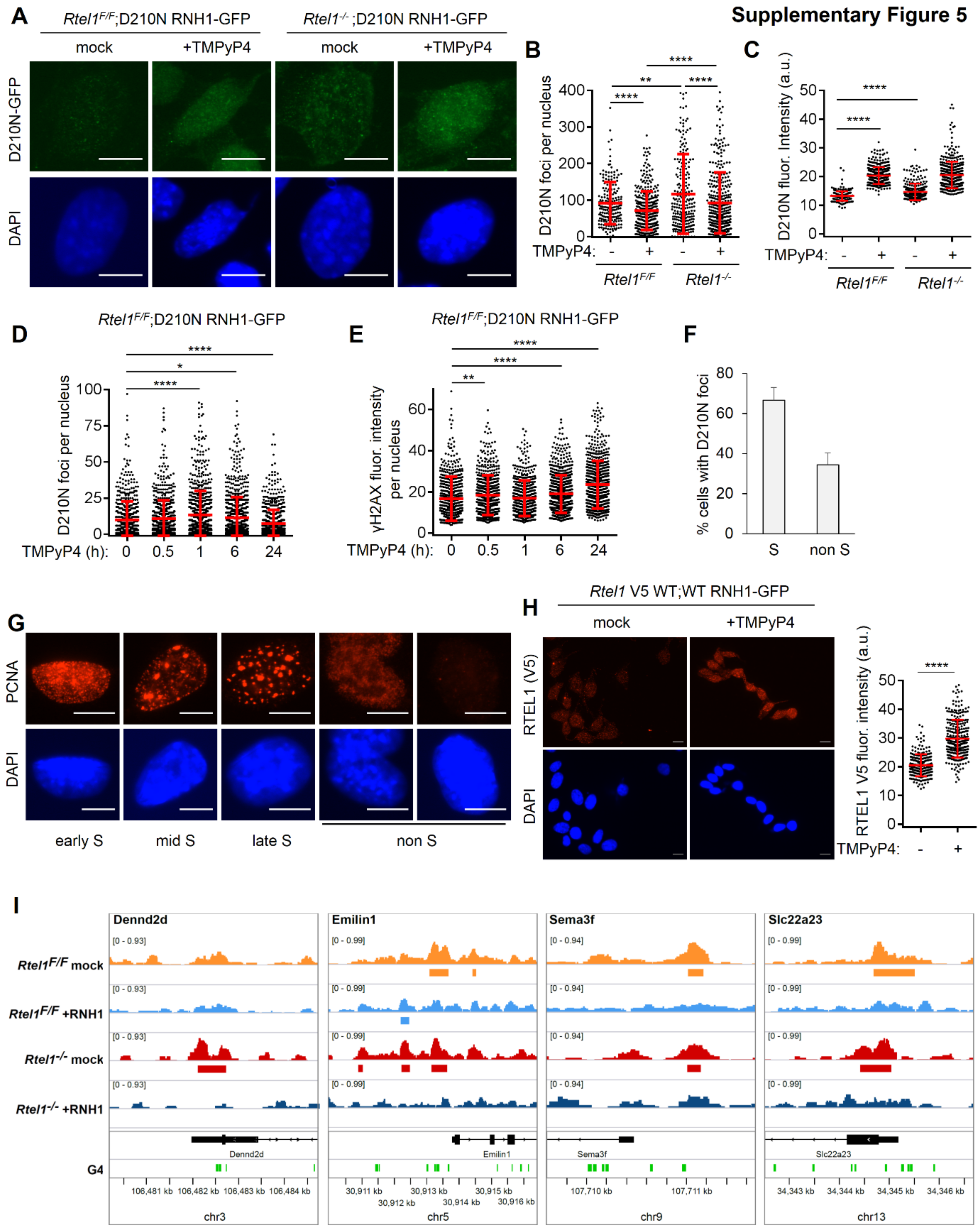

Supplementary Figure 5. TMPyP4-induced recruitment of RNaseH1^{D210N}-GFP foci precedes DNA damage. Related to Figure 5.

(A) *Rtel1$^{F/F}$*;D210N RNH1-GFP MEFs were infected with RFP or iCre-RFP adenovirus, after 48 h doxycycline was added, after 24 h TMPyP4 was added, cells were incubated for 24 h, pre-extracted, fixed and immunostained for GFP. Representative images the immunostaining.

(B) Quantification of number of RNaseH1^{D210N}-GFP foci per nucleus of (A). Data are represented as mean ± SD (n=2).

(C) Quantification of fluorescence intensity of RNaseH1^{D210N}-GFP per nucleus of (A). Data are represented as mean ± SD (n=2).

(D) *Rtel1$^{F/F}$*;D210N RNH1-GFP MEFs were treated with TMPyP4 for 0.5, 1, 6 and 24 h pre-extracted, fixed and immunostained for GFP. Quantification of number of RNaseH1^{D210N}-GFP foci per nucleus. Data are represented as mean ± SD (n=4).

(E) *Rtel1$^{F/F}$*;D210N RNH1-GFP MEFs were treated with TMPyP4 for 0.5, 1, 6 and 24 h pre-extracted, fixed and immunostained for γH2AX. Quantification of γH2AX fluorescence intensity per nucleus. Data are represented as mean ± SD (n=3).

(F) *Rtel1$^{F/F}$*;D210N RNH1-GFP MEFs were treated with TMPyP4 for 1 h pre-extracted, fixed with 4% PFA, permeabilized with methanol and immunostained for GFP and PCNA. Quantification of number of cells that contain TMPyP4-induced RNaseH1^{D210N}-GFP foci and are in S or non S phase according to PCNA staining. Data are represented as mean ± SD (n=2).

(G) Representative images of PCNA staining.

(H) *Rtel1* V5 WT;WT RNH1-GFP MEFs were treated with TMPyP4 for 24 h, pre-extracted, fixed and immunostained for V5. Left panel: Representative images of RTEL1-V5 immunostaining. Right panel: Quantification of fluorescence intensity of RTEL1-V5 per nucleus.

(I) *Rtel1$^{F/F}$*;WT RNH1-GFP MEFs were infected with GFP or Cre-GFP adenovirus, after 48 h doxycycline was added and cells were collected after 48 h and used for R-loop detection with DRIP-seq. Genome browser plots of normalised read coverage, called peaks of DRIP-Seq and associated predicted G4 structures in four different genomic locations.

P values determined by unpaired T-test $*p<0.05$, $**p<0.01$, $***p<0.001$, $****p<0.0001$. Scale bars: 10 μm.
